# Role of Traditional Chinese Medicine in Treating Severe or Critical COVID-19: A Systematic Review of Randomized Controlled Trials and Observational Studies

**DOI:** 10.3389/fphar.2022.926189

**Published:** 2022-07-15

**Authors:** Mengting Li, Hongfei Zhu, Yafei Liu, Yao Lu, Minyao Sun, Yuqing Zhang, Jiaheng Shi, Nannan Shi, Ling Li, Kehu Yang, Xin Sun, Jie Liu, Long Ge, Luqi Huang

**Affiliations:** ^1^ Department of Social Medicine and Health Management, School of Public Health, Lanzhou University, Lanzhou, China; ^2^ Evidence Based Social Science Research Centre, School of Public Health, Lanzhou University, Lanzhou, China; ^3^ Evidence Based Nursing Centre, School of Nursing, Lanzhou University, Lanzhou, China; ^4^ Department of Health Research Methods, Evidence, and Impact, McMaster University, Hamilton, ON, Canada; ^5^ CEBIM (Center for Evidence Based Integrative Medicine)-Clarity Collaboration, Guang’ Anmen Hospital, China Academy of Chinese Medical Sciences, Beijing, China; ^6^ Institute of Acupuncture and Moxibustion, China Academy of Chinese Medical Sciences, Beijing, China; ^7^ Nottingham Ningbo GRADE Center, The University of Nottingham Ningbo, Ningbo, China; ^8^ China Center for Evidence Based Traditional Chinese Medicine, China Academy of Chinese Medical Sciences, Beijing, China; ^9^ Department of Emergency, Guang’ Anmen Hospital, China Academy of Chinese Medical Sciences, Beijing, China; ^10^ Chinese Evidence-Based Medicine Center, West China Hospital, Sichuan University, Chengdu, China; ^11^ Evidence-Based Medicine Center, School of Basic Medical Sciences, Lanzhou University, Lanzhou, China; ^12^ WHO Collaborating Center for Guideline Implementation and Knowledge Translation, Lanzhou, China; ^13^ Key Laboratory of Evidence Based Medicine and Knowledge Translation of Gansu Province, Lanzhou, China; ^14^ Department of Oncology, Guang’ Anmen Hospital, China Academy of Chinese Medical Sciences, Beijing, China; ^15^ National Resource Center for Chinese Materia Medica, China Academy of Chinese Medical Sciences, Beijing, China

**Keywords:** traditional Chinese medicine, COVID-19, systematic review, effects, safety

## Abstract

**Background:** The coronavirus disease 2019 (COVID-19) continues to spread globally. Due to the higher risk of mortality, the treatment of severe or critical patients is a top priority. Traditional Chinese medicine (TCM) treatment has played an extremely important role in the fight against COVID-19 in China; a timely evidence summary on TCM in managing COVID-19 is crucial to update the knowledge of healthcare for better clinical management of COVID-19. This study aimed to assess the effects and safety of TCM treatments for severe/critical COVID-19 patients by systematically collecting and synthesizing evidence from randomized controlled trials (RCTs) and observational studies (e.g., cohort).

**Methods:** We searched nine databases up to 19th March 2022 and the reference lists of relevant publications. Pairs of reviewers independently screened studies, extracted data of interest, and assessed risk of bias. We performed qualitative systematic analysis with visual presentation of results and compared the direction and distribution of effect estimates for each patient’s important outcome. We performed sensitivity analyses to observe the robustness of results by restricting analysis to studies with low risk of bias.

**Results:** The search yielded 217,761 records, and 21 studies (6 RCTs and 15 observational studies) proved eligible. A total of 21 studies enrolled 12,981 severe/critical COVID-19 patients with a mean age of 57.21 years and a mean proportion of men of 47.91%. Compared with usual supportive treatments, the effect estimates of TCM treatments were consistent in direction, illustrating that TCM treatments could reduce the risk of mortality, rate of conversion to critical cases, and mechanical ventilation, and showed significant advantages in shortening the length of hospital stay, time to viral clearance, and symptom resolution. The results were similar when we restricted analyses to low-risk-bias studies. No serious adverse events were reported with TCM treatments, and no significant differences were observed between groups.

**Conclusion:** Encouraging evidence suggests that TCM presents substantial advantages in treating severe/critical COVID-19 patients. TCM has a safety profile that is comparable to that of conventional treatment alone. TCMs have played an important role in China’s prevention and treatment of COVID-19, which sets an example of using traditional medicine in preventing and treating COVID-19 worldwide.

## Introduction

Coronavirus disease 2019 (COVID-19) is an acute infectious disease caused by the severe acute respiratory syndrome coronavirus virus 2 (SARS-CoV-2), which has become the most widespread global pandemic that human beings have encountered in the past 100 years (WHO, 2020) and continues to threaten human life and health. As of March 8, 2022, more than 433 million patients have been affirmed worldwide; of those, more than 5.9 million have died (WHO, 2022a).

According to the World Health Organization (WHO) severity definitions, COVID-19 patients are categorized into non-severe, severe, and critical (Agarwal et al., 2020). In the clinical practice, severe or critical patients have the top priorities in the treatment of COVID-19 because of a higher risk of mortality. Despite global efforts to identify effective therapeutic strategies for severe or critical COVID-19, only systemic corticosteroids, interleukin 6 (IL-6) receptor blockers (tocilizumab or sarilumab), or baricitinib combined with corticosteroids were strongly recommended by the WHO after evaluating more than 200 drugs involved in more than 400 randomized controlled trials (RCTs).

Integrated traditional Chinese medicine (TCM) and western medicine has played an extremely important role in the fight against COVID-19 in China during the pandemic (Ge et al., 2021). According to the WHO Report on the evaluation of TCM in the treatment of COVID-19, TCM has been demonstrated to be beneficial for mild-to-moderate COVID-19 patients; however, they did not make a conclusion for severe/critical patients because only one RCT was included for evaluation (WHO, 2022b). At present, encouraging evidence has been documented that TCM presented substantial benefits for severe or critical patients such as reducing the risk of mortality (Chen G. et al., 2020; Wang et al., 2021a; Sun et al., 2021; Zhang L. et al., 2021; Zhou et al., 2021) and rate of conversion to critical cases (Chan et al., 2020; Xu Z. et al., 2020), shortening time of nucleic acid conversion (Wang et al., 2021b; Hu H. et al., 2021), and improving the local and systemic inflammatory response (Chen J. et al., 2020; Fan et al., 2020; Runfeng et al., 2020). Given there is still no globally uniform therapeutic strategy for severe or critical COVID-19 patients, a timely evidence summary is crucial to update the knowledge of healthcare for better clinical treatment of COVID-19.

To provide trustworthy evidence to elaborate on the role of TCM against COVID-19, we conducted a systematic review with a rigorous methodology to comprehensively summarize the efficacy and safety of TCM in the treatment of severe/critical COVID-19 patients by systematically collecting available evidence from RCTs and observational studies.

## Methods

This study was conducted and reported following the Preferred Reporting Items for Systematic Reviews and Meta-Analyses (PRISMA) checklist (Page et al., 2021).

### Eligibility Criteria

We included studies that met the following criteria: 1) patients with confirmed severe or critical COVID-19 according to the national or international recognized diagnosis standard, aged 18 years or older; studies that included patients with both non-severe and severe or critical COVID-19 were eligible if more than 80% patients were severe and/or critical; and studies that included information of severe or critical patients that could be extracted from a subgroup-analysis also were eligible. 2) Comparison: on the basis of usual supportive treatment, treatment with TCM versus without TCM. We did not limit the form of Chinese medicine; granules, decoction, and injections were all considered to be included. 3) Outcomes: we decided the outcomes of interest according to a living network meta-analysis published in *BMJ* (Siemieniuk et al., 2020) and a core outcome sets of COVID-19 (Jin et al., 2020) mainly including: a. clinical efficacy (e.g., mortality, length of hospital stay, and rate of mechanical ventilation), b. clinical symptoms (e.g., fever, cough, expectoration, and tiredness), c. laboratory indicators (e.g., lymphocyte percentage, white blood cell (WBC) count, C-reactive protein (CRP), and tumor necrosis factor-α (TNF-a)), d. adverse events (e.g., nausea and vomit, diarrhea, and abnormal liver function). 4) Study types: RCTs and observational studies (e.g., cohort study and historical control study).

We excluded studies that mainly did not report information about the ethical approval, and the study design was protocol, case report, case report series, cross-sectional study, and controlled before-after study.

### Literature Search

Systematic searches were performed under the guidance of an experienced librarian of nine databases from December 2019 to 19th March 2022, including PubMed, Embase, Cochrane Central Register of Controlled Trials (CENTRAL), Web of Science, China National Knowledge Infrastructure (CNKI), WanFang Database, Chinese Biomedical Literature Database (CBM), China Science and Technology Journal Database (VIP), and the L-OVE COVID-19 Repository. We employed an extremely sensitive search strategy, which only included search terms related to disease (COVID-19) and study design, without restrictions on publication language and interventions. (see Additional file 1: Supplementary Table S1 for detailed search strategies). We also tracked the references of relevant publications.

### Study Selection

We used EndNote X8.0 to manage the initial searched records. After removing duplicate records, the remaining records were imported into online reference management software Rayyan (Ouzzani et al., 2016). In total, teams of 2 reviewers (LMT and XXL, LYF and ZHF, and PB and LHH), following training and calibration exercises, independently screened the title and abstracts of each record and downloaded the full text of potentially eligible studies for further reviewing to determine the final eligibility. Any conflict was resolved through discussion.

### Data Extraction

For each eligible trial, teams of two reviewers (LMT and ZHF, SMY and LY, and LYF and TC), following training and calibration exercises, extracted data independently using a standardized, pilot-tested data extraction form. We used a data extraction form to collate data from the included studies. We extracted the following from the studies: general information (first author, year of publication, trial registration, journal, timeline of patient recruitment, and recruitment location), characteristics of patients (age, patient type, sample size, sex, and complications), details of interventions (specific treatment measures, dose, components of Chinese herbal medicine used, and treatment duration), type of study design, and outcomes of interest.

### Risk of Bias Assessment

For each eligible RCT, teams of two reviewers (LMT and ZHF, SMY and LY, and LYF and TC), following training and calibration exercises, independently assessed the risk of bias using a modification of the Cochrane tool for assessing risk of bias in randomized clinical trials (RoB 2.0) (Sterne et al., 2019; MAGIC 2022a) from the following six domains: bias from the randomization process generated, bias due to deviations from the intended intervention, bias due to missing data, bias in measurement of the outcome, bias in selection of the reported results, and bias due to other sources (e.g., consistency between the registration information and the final report and completeness of the report).

The risk of bias of each eligible cohort study was assessed using a revised Risk of Bias in Non-Randomized Studies of Interventions (ROBINS-I) (Sterne et al., 2016; MAGIC 2022b) from the following six domains: bias due to confounding, bias in selection of participants into the study, bias from the interventions, bias due to missing data, bias due to measurement of the outcome, and bias in selection of the reported results.

Based on the aforementioned criteria for identifying the risk of bias, eligible studies were categorized into four groups: 1) low risk of bias, 2) probably low risk of bias, 3) probably high risk of bias, and 4) high risk of bias. Reviewers resolved discrepancies by discussion and, when not possible, with adjudication by a third party (GL). Detailed guidance for assessment of risk of bias was presented in Additional file 1: Supplementary Table S2.

### Evidence Synthesis

When there were two or more eligible studies with the same study design, intervention, and controls, a meta-analysis was performed using Review Manager software (RevMan, version 5.3, Copenhagen: The Nordic Cochrane Centre, The Cochrane Collaboration, 2014) (Wang Q. et al., 2021). If the aforementioned requirements cannot be met, a qualitative systematic analysis would be conducted based on the data of RCT and observational studies, respectively. For dichotomy, we calculated the risk ratio (RR) with corresponding 95% confidence interval (95%CI). For continuous outcomes, we calculated mean difference (MD) with 95%CI. We directly used effect measures reported in observational studies if they reported the results with adjustment of confounders. Findings from each individual study were graphically presented using the effect of direction plot, which showed the benefits and harms of the included studies for each outcome. The risk of bias for each outcome was shown in different color: green for low risk of bias and red for high risk of bias. The direction of the arrow represented the benefits (upwards) and harms (downwards) impact. If the arrow was closed and filled, the effect of intervention was statistically significant; the hollow arrow indicated that there was no significant difference. We drew clustered bar charts to represent the proportion or time for both treatment/exposure and control/non-exposure groups, and effect measures were calculated. The color of the bars shows the risk of bias. In addition, an effect direct plot and clustered bar charts also were used to represent type of patients (proportion of severe or critical patients), type of TCM treatments, study design, and sample size. We compared the direction and distribution of effect estimates of different TCM treatments across studies to show the benefit and harm of TCM compared with usual supportive treatment. We restricted analyses to studies with low risk of bias to observe the robustness of results. We considered that the results were robust if more than half of studies were low risk of bias for each outcome. If the number of studies was sufficient, we also performed subgroup analyses by severity of patients, type of TCM treatments, age, and comorbidities. A radar chart was used to show the rate of common adverse reactions in included studies, and the incidence rate of each adverse reaction corresponded to a coordinate axis respectively.

## Results

**FIGURE 1:**
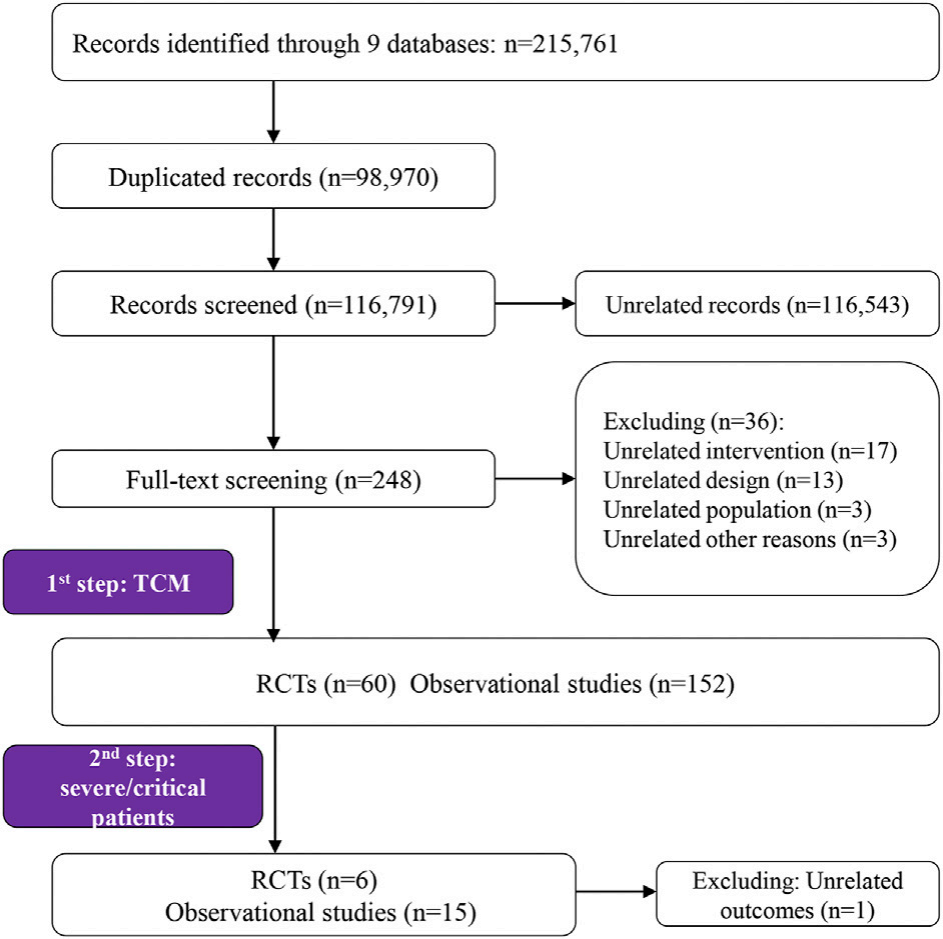
Study selection PRISMA flow chart.

Our searches yielded 215,761 unique studies. Of the total, 248 were potentially eligible and for further full-text review. We identified 212 studies related to TCM intervention in the first step and further restricted to studies that focused on severe and/or critical patients, in which 21 studies (six RCTs and 15 observational studies) proved eligible (Figure 1).

### Characteristics of Included Studies

Twenty-one studies enrolled 12,981 patients (446 patients in RCTs and 12,531 patients in observational studies), with a mean age of 57.21 years and a mean proportion of men of 47.91%. Thirteen studies (3 RCTs (Hu F. et al., 2021; Liu S. T. et al., 2021; Luo et al., 2021) and 10 observational studies (Qin et al., 2020; Wang et al., 2021a; Wang et al., 2021b; Feng et al., 2021; Hu H. et al., 2021; Liu X. S. et al., 2021; Shu et al., 2021; Sun et al., 2021; Zhang L. et al., 2021; Xiong et al., 2022)) reported on comorbidities; the most common comorbidities were diabetes, hypertension, cardiovascular disease or coronary heart disease, and respiratory conditions. All patients recruited were from China, of which 76.19% were in the Hubei Province (Chen G. et al., 2020; Wang et al., 2021a; Wang et al., 2021b; Feng et al., 2021; Hu F. et al., 2021; Hu H. et al., 2021; Huang et al., 2021; Liu, 2021; Liu S. T. et al., 2021; Luo et al., 2021; Shu et al., 2021; Sun et al., 2021; Zhang L. et al., 2021; Zhang and Pan, 2021; Zhou et al., 2021; Xiong et al., 2022), and the time was concentrated at the beginning of the pandemic outbreak (January to May 2020). Fourteen studies (4 RCTs (Hu F. et al., 2021; Liu S. T. et al., 2021; Luo et al., 2021; Zhou et al., 2021) and 10 observational studies (Zhao et al., 2020b; Chen G. et al., 2020; Wang et al., 2021a; Wang et al., 2021b; Feng et al., 2021; Hu H. et al., 2021; Huang et al., 2021; Shu et al., 2021; Sun et al., 2021; Zhang L. et al., 2021)) were published in English, and 8 (5 RCTs (Wen et al., 2020; Hu F. et al., 2021; Liu S. T. et al., 2021; Luo et al., 2021; Zhou et al., 2021) and 3 observational studies (Chen G. et al., 2020; Liu X. S. et al., 2021; Sun et al., 2021)) were registered prospectively. Additional file 1: Supplementary Table S3 presents the detailed study characteristics.

Twenty-one studies involved 15 TCM treatments, including Xuebijing injection (XBJ), Huashi Baidu granules (HSBD), Chansu injection (CS), Shenghuang granules (SH), Qingfei Paidu decoction (QFPD), Reduning injection (RDN), Yidu-toxicity blocking lung (YDZF), Xiyanping injecton (XYP), Shenmai injection (SM), Hejie Shenshi decoction (HJSS), Gengzi No.3 recipe (GZ 3), Mahuang Liu Jun Tang (MHLJ), Chaihu Jiedu granules (CHJD), Fuzheng Jiufei granules (FZJF), and semi-individualized TCM. The usual supportive treatment was performed mainly according to the treatment regimens recommended by the “Diagnosis and Treatment Protocol for COVID-19” (3rd to 7th Edition), including usual care, antivirus, and antibacterial treatment measures. Additional file 1: Supplementary Table S4 presents the detailed treatment and control information.

### Risk of Bias in Included Studies

**FIGURE 2:**
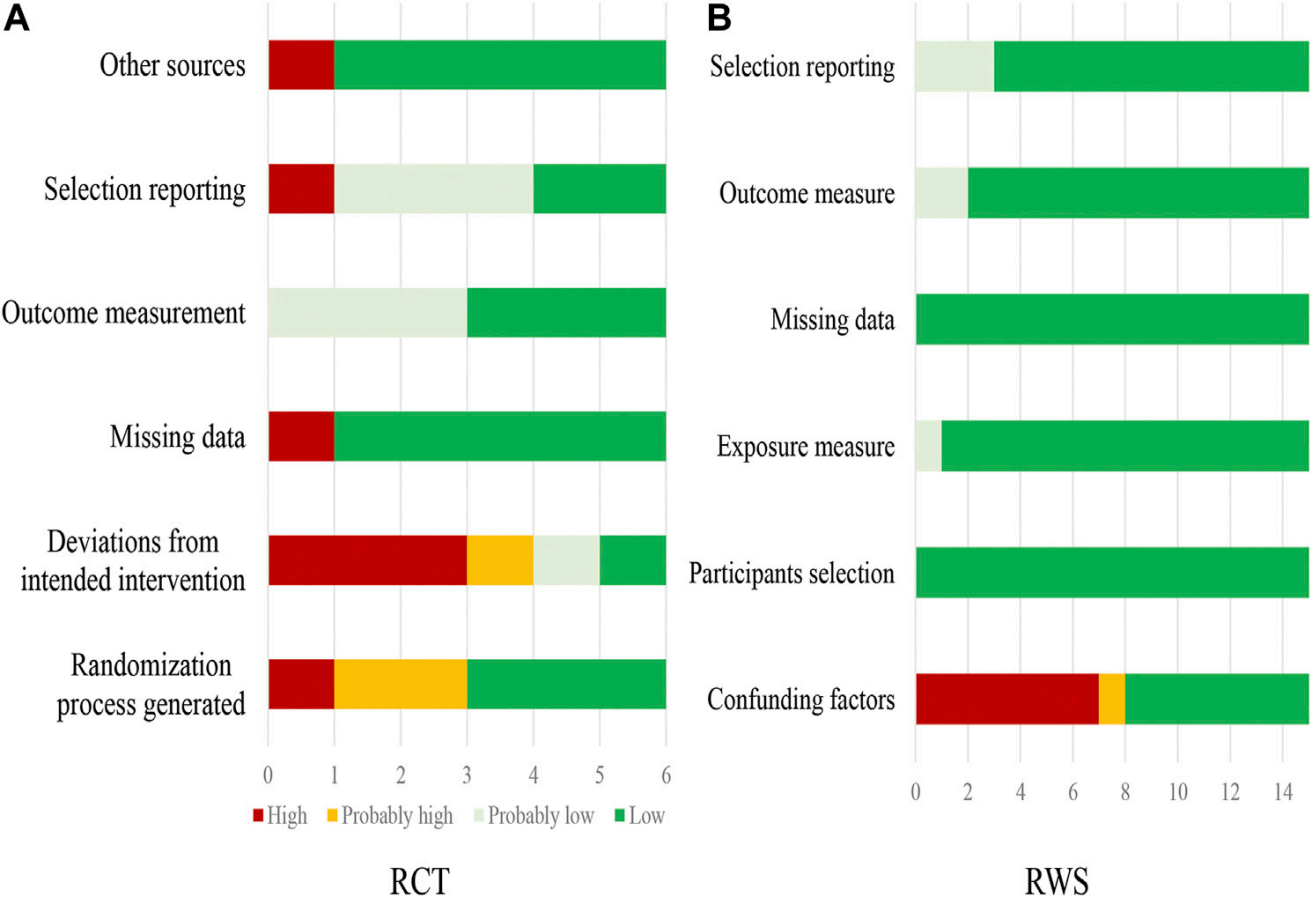
Risk of bias of included RCTs **(A)** and observational studies **(B)**.

Additional file 1: Supplementary Table S5 presents the assessment of risk of bias of single RCTs. A summary of risk of bias of six RCTs is presented in Figure 2A. Only two RCTs (Luo et al., 2021; Zhou et al., 2021) were judged at low or probably low risk of bias in all domains. All other RCTs had probably high or high risk of bias in at least one of the domains. The limitations mainly were from the concealment of random sequence number assignment and unblinding.

Additional file 1: Supplementary Table S6 presents the assessment of risk of bias of single observational studies. A summary of risk of bias of 15 observational studies is presented in Figure 2B. Seven observational studies(Chen G. et al., 2020; Wang et al., 2021a; Hu H. et al., 2021; Liu X. S. et al., 2021; Shu et al., 2021; Sun et al., 2021; Zhang L. et al., 2021) were judged at low or probably low risk of bias in all domains. Eight eight studies (Zhao et al., 2020b; Qin et al., 2020; Wang et al., 2021b; Chen L. et al., 2021; Huang et al., 2021; Zhang and Pan, 2021; Zhou et al., 2021; Xiong et al., 2022) had probably high or high risk of bias in at least one of the domains. The limitations mainly were un-adjustment of confounding factors.

### Effects of TCM Interventions

**FIGURE 3:**
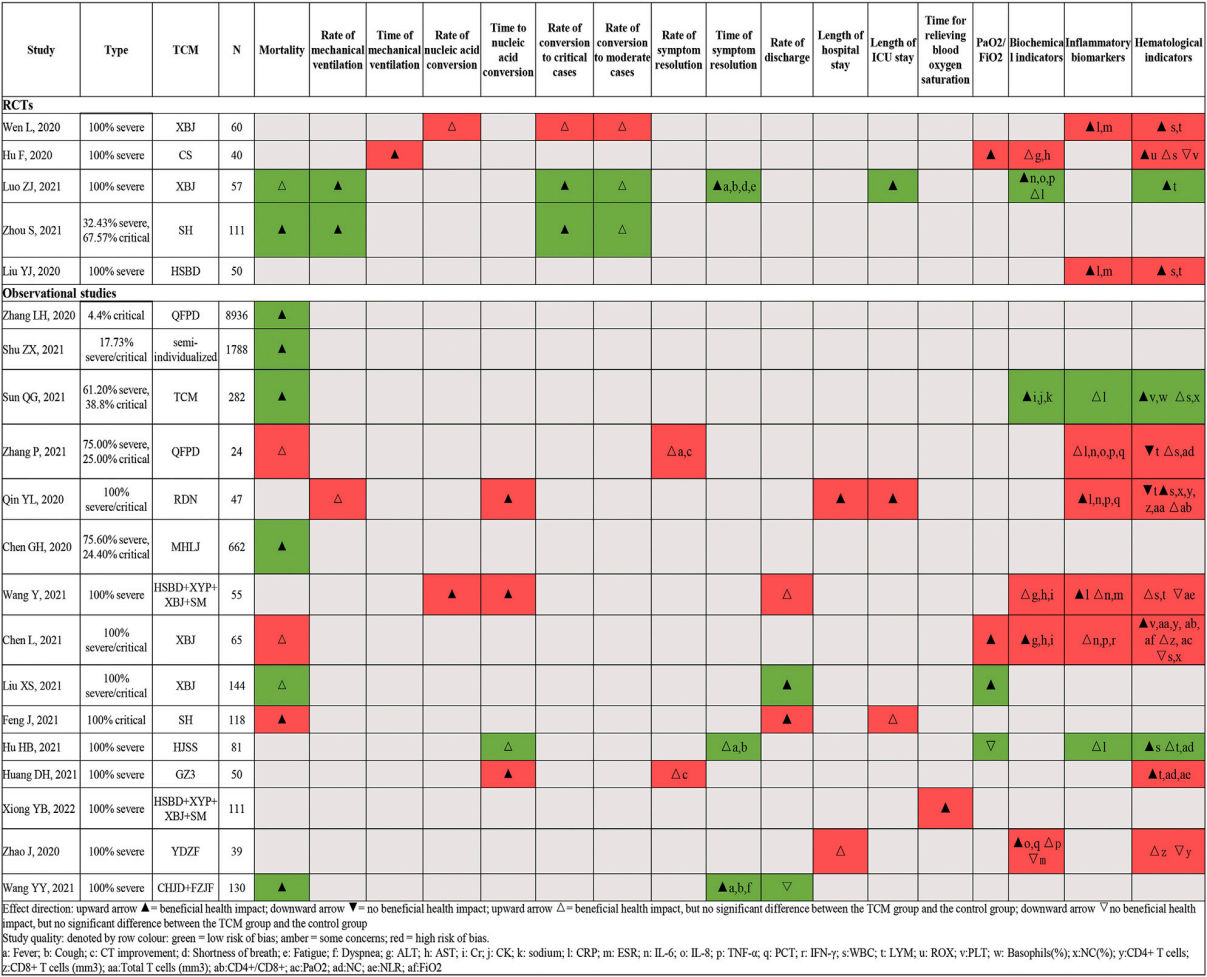
Effect direction plot summarizing direction of health impacts from each single study.

**FIGURE 4:**
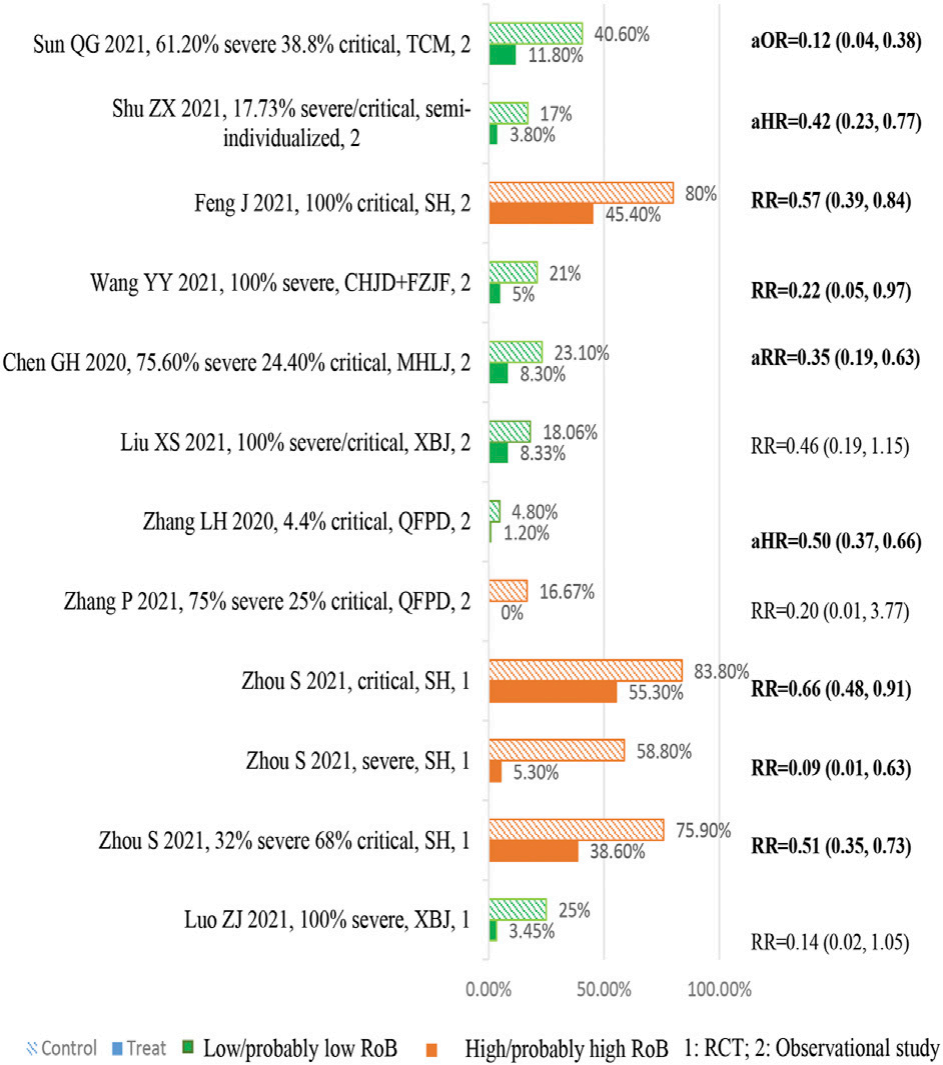
Summary chart of mortality.

**FIGURE 5:**
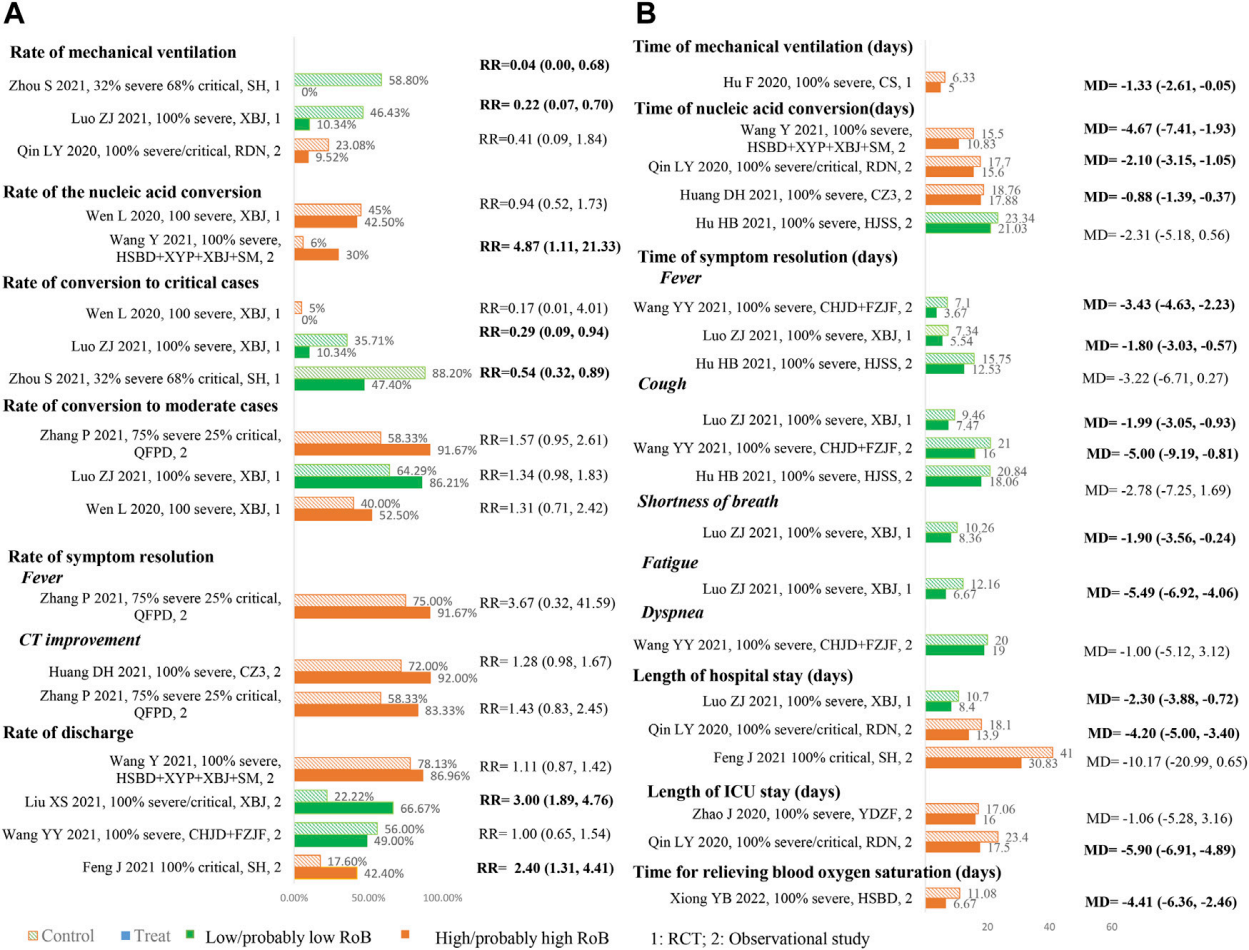
Summary of dichotomous **(A)** and continuous **(B)**.

Figure 3 is an effect direction plot that presents the benefit and harm of included studies for each outcome. Although about half of the results were from studies with high risk of bias, it clearly showed that almost all studies had beneficial health impact on the outcomes of interest. Figure 4 and Figure 5 show the proportions or times for treatment/exposure and control/non-exposure group and the calculated effect measures for all outcomes. Figure 5 presents the dichotomous outcomes on the left and the continuous outcomes on the right. Additional file 1: Supplementary Tables S5–S14 presents the detailed outcome data of intervention group *vs*. control group, and effect estimates with 95% confidence interval (CI).

#### Mortality

Ten studies (two RCTs(Luo et al., 2021; Zhou et al., 2021) and eight observational studies (Chen G. et al., 2020; Wang et al., 2021a; Feng et al., 2021; Liu X. S. et al., 2021; Shu et al., 2021; Sun et al., 2021; Zhang L. et al., 2021; Zhang and Pan, 2021)) enrolling 12,252 severe/critical patients reported mortality (Figure 4). Compared with usual supportive treatments, the effect estimates of all TCM treatments were consistent in direction, illustrating that TCMs had more benefits. Of them, seven studies showed statistical differences in reducing the risk of mortality. One study reported accumulate survival rate and showed that TCM have a potentially higher survival rate although no significant difference was observed. A national retrospective registry study enrolled 8,936 mixed patients (including severe and non-severe) to explore the association between QFPD (Zhang L. et al., 2021) use and in-hospital mortality, and their subgroup analyses showed that there were no significant subgroup effects for sex and age (P _interaction_ = 0.67, 0.86, respectively); however, a larger reduction in the risk of mortality was found in patients without prior medical history/comorbidities status (P _interaction_ = 0.02). Another cohort study (Chen G. et al., 2020) enrolled 662 patients, and a multivariate logistic regression analysis showed that the risk of mortality increased by 5.0% with every 1 year of age increase.

#### Rate/Duration of Mechanical Ventilation

Three studies (two RCTs (Luo et al., 2021; Zhou et al., 2021) and one observational study (Qin et al., 2020)) enrolling 215 severe/critical patients reported the rate of mechanical ventilation (Figure 5 A). All studies showed consistent direction that TCM treatments could reduce the mechanical ventilation rate, and both RCTs showed significant differences between TCM treatments and usual supportive treatment. One RCT (Hu F. et al., 2021) including 40 severe COVID-19 patients showed that TCM could significantly shorten the duration of mechanical ventilation compared to usual supportive treatment (Figure 5 B).

#### Rate/Duration of Nucleic Acid Conversion

Two studies (one RCT (Wen et al., 2020) and one observational study (Wang et al., 2021b)) involving 111 severe patients reported this outcome (Figure 5 A). Evidence from RCT (Wen et al., 2020) did not show significant improvement in increasing the rate of nucleic acid conversion at 7 days; however, an observational study (Wang et al., 2021b) showed that TCM treatment could significantly increase the rate of nucleic acid conversion compared to usual supportive treatment.

Four observational studies (Hu H. et al., 2021; Huang et al., 2021; Shu et al., 2021; Xiong et al., 2022) enrolling 233 severe/critical patients reported the time to the nucleic acid conversion (Figure 5 B). Compared with usual supportive treatments, the effect estimates of all TCM treatments were consistent in direction, illustrating that TCMs had more benefits. Of them, three studies (Huang et al., 2021; Shu et al., 2021; Xiong et al., 2022) showed statistical differences in the shortening time of nucleic acid conversion, which ranged from 0.88 to 5.67 days.

#### Rate of Conversion to Moderate/Critical Cases

Four studies (three RCTs(Wen et al., 2020; Luo et al., 2021; Zhou et al., 2021) and one observational study (Xiong et al., 2022)) enrolling 339 severe/critical patients reported the rate of conversion to critical cases (Figure 5 A). Three studies (two RCTs (Wen et al., 2020; Luo et al., 2021) and one observational study (Zhang and Pan, 2021)) enrolling 141 severe/critical patients reported the rate of conversion to moderate cases (Figure 5 A). All studies showed consistent direction that TCM treatments could reduce the rate of conversion of severe to critical cases and increase the rate of conversion of severe to moderate cases. Two RCTs (Wen et al., 2020; Luo et al., 2021) showed statistical differences in reducing rate of conversion of severe to critical cases.

#### Rate/Time of Symptom Resolution

Two observational studies (Huang et al., 2021; Zhang and Pan, 2021) enrolling 74 severe/critical patients reported the rate of symptom resolution, and three studies (one RCT(Luo et al., 2021) and two observational studies (Wang et al., 2021a; Hu H. et al., 2021)) enrolling 268 severe patients reported the time of symptom resolution (Figure 5 B). All studies showed consistent direction that TCM treatments could increase the rate of symptom resolution and shorten the time of symptom resolution. Two studies (Wang et al., 2021a; Luo et al., 2021) showed statistical differences in shortening the time of symptom resolution.

#### Discharge Rate and Length of Hospital Stay

The rate of discharge was reported in four observational studies (Wang et al., 2021a; Wang et al., 2021b; Feng et al., 2021; Liu X. S. et al., 2021) on 447 severe/critical patients (Figure 5 A). Compared with usual supportive treatments, the direction of effect estimates of TCM treatments were consistent in reducing rate of discharge, illustrating that TCM had more advantages. Four studies (one RCT (Luo et al., 2021) and three observational studies (Zhao et al., 2020b; Qin et al., 2020; Feng et al., 2021)) with four TCM enrolled 261 severe/critical patients reported the length of hospital/ICU stay (Figure 5 B). Compared with usual supportive treatment, RDN significantly shortened length of hospital stay; XBJ and RDN also showed advantages in shortening the length of ICU stay.

#### Biochemical Indicators

We divided laboratory indicators into biochemical, inflammatory biomarkers, coagulation, and hematologic. The specific indicators included are detailed in Supplementary Table S13. Four studies (one RCT (Hu F. et al., 2021) and four observational studies (Qin et al., 2020; Wang et al., 2021b; Chen L. et al., 2021; Sun et al., 2021)) enrolling 489 severe/critical patients reported biochemical indicators. The results showed that after XBJ (Chen L. et al., 2021) treatment, alanine transaminase (ALT) and aspartate aminotransferase (AST) were significantly higher than those before treatment and better than usual supportive treatment, with statistical difference. TCMD (Sun et al., 2021) was statistically better than the non-exposed group in restoring serum creatinine (Cr) and creatine kinase (CK) to normal values.

#### Inflammatory Biomarkers

Nine studies (three RCTs (Wen et al., 2020; Liu, 2021; Luo et al., 2021) and six observational studies (Qin et al., 2020; Wang et al., 2021b; Chen L. et al., 2021; Hu H. et al., 2021; Sun et al., 2021; Zhang and Pan, 2021)) enrolling 721 severe/critical patients reported Inflammatory biomarkers (Supplementary Table S13). Compared with usual supportive treatment, the levels of procalcitonin (PCT), CRP, IL-4, IL-6, IL-10, IL-17, TNF-α, and interferon-γ (IFN-γ) in the RDN (Qin et al., 2020) group were significantly decreased, with statistical significance. The levels of CRP and erythrocyte sedimentation rate (ESR) in the HSBD group (Liu, 2021) were significantly lower than those in the usual supportive treatment group, and there was a statistical difference. Compared with usual supportive treatment, CRP, ESR, IL-6, IL-8, and TNF-α levels in the XBJ groups (Wen et al., 2020; Luo et al., 2021) significantly decreased after treatment, and the difference between the two groups was statistically significant.

#### Hematological Indicators

Eleven studies (four RCTs(Wen et al., 2020; Hu F. et al., 2021; Liu, 2021; Luo et al., 2021) and seven observational studies (Qin et al., 2020; Wang et al., 2021b; Chen L. et al., 2021; Hu H. et al., 2021; Huang et al., 2021; Sun et al., 2021; Zhang and Pan, 2021)) enrolling 811 severe/critical patients reported hematological indicators (Supplementary Table S13). Compared with the usual supportive treatment group, TCM had advantages in the return of WBC, neutrophil count (NC), lymphocyte (LYM) count, LYM (%), NC/LYM (NLR), T-LYM count, CD4^+^ cells, CD8^+^ cells, platelet count (PLT), and basophils (%) returned to normal values, and the ROX index (ROX = SpO2/(FiO2*RR)) was significantly higher than that of the usual supportive treatment group.

#### Other Efficacy Outcomes

Four studies (one RCT(Hu F. et al., 2021) and three observational studies (Chen L. et al., 2021; Hu H. et al., 2021; Liu X. S. et al., 2021)) enrolling 330 severe/critical patients reported PaO_2_/FiO_2_ (Supplementary Table S14). CS (Hu F. et al., 2021) and XBJ (Chen L. et al., 2021; Liu X. S. et al., 2021) could improve PaO_2_/FiO_2_ compared to usual supportive treatment group. In an observational study (Xiong et al., 2022) of 111 severe patients, the time for relieving blood oxygen saturation in HSBD was better than that that in usual supportive treatment, with a statistically significant difference (Figure 5 B).

A study enrolling 55 severe patients reported coagulation index: D-dimer, and the results showed that there was no statistical difference between TCM and usual supportive treatment groups (Supplementary Table S13).

One RCT (Wang et al., 2021b) enrolling 128 severe patients showed that qigong exercise and an acupressure rehabilitation program during the treatment period could significantly shorten length of hospital stay and improve lung function and symptoms.

### Safety of TCM Interventions

**FIGURE 6:**
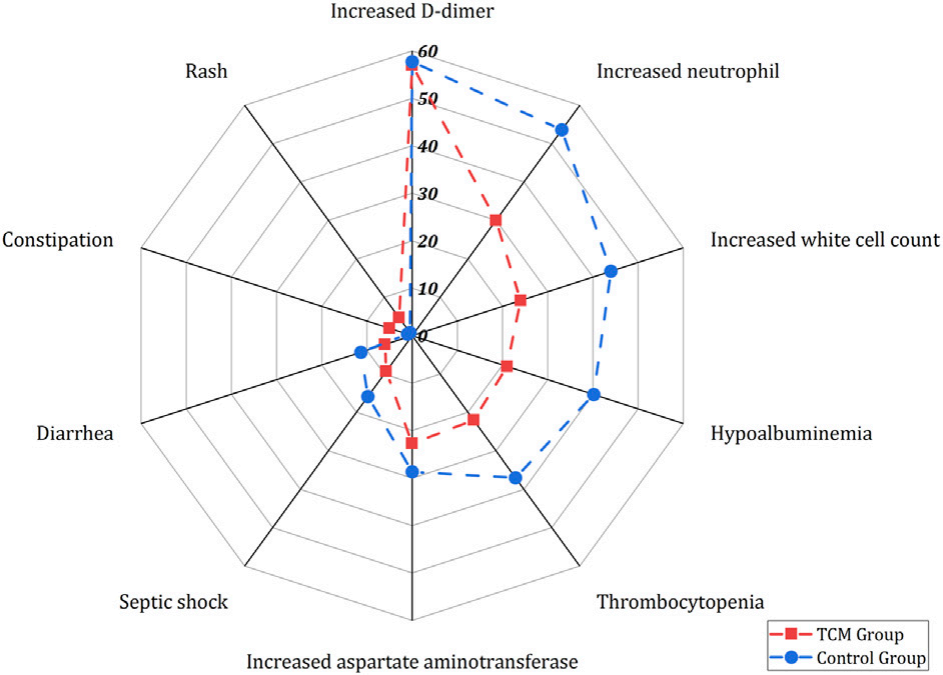
Summary of adverse events.

Eight studies (four RCTs(Hu F. et al., 2021; Liu, 2021; Luo et al., 2021; Zhou et al., 2021) and four observational studies (Wang et al., 2021a; Wang et al., 2021b; Liu X. S. et al., 2021; Zhang L. et al., 2021)) including 9,482 patients reported 76 types of adverse events (Figure 6, see Additional file 1: Supplementary Table S15 for more details). Regarding the gastrointestinal bleeding and prolonged coagulation time, there exist statistical differences between the HSBD group (Liu, 2021) and the usual supportive treatment group. Compared with the usual supportive treatment, the XBJ group (Luo et al., 2021) showed statistical difference in reducing the incidence of acute respiratory distress syndrome and septic shock. The results of the SH group (Zhou et al., 2021) showed significant difference in reducing the incidence of hypoalbuminemia, increased blood glucose, thrombocytopenia, increased total bilirubin, increased white cell count, increased blood urea nitrogen, increased neutrophil, increased aspartate aminotransferase, diarrhea, increased serum potassium, respiratory failure or acute respiratory distress syndrome, cardiopulmonary failure, cardiac arrest, thrombocytopenia, and increased D-dimer and multiple organ dysfunction syndrome when compared with the usual supportive treatment group. Commonly, patients in the TCM group had a lower incidence rate among the aforementioned adverse reactions.

Adverse reactions reported in two or more studies included rash, constipation, diarrhea, septic shock, increased aspartate aminotransferase, thrombocytopenia, hypoalbuminemia, increased white cell count, increased neutrophil, and increased D-dimer. Except for rash and constipation, the overall incidence of adverse reactions in the TCM group was lower than that in the usual supportive treatment group.

## Discussion

### Main Findings

During the COVID-19 pandemic, China completely utilized the unique advantages of TCM in treating pandemics and combined it with western medicine to make great contributions to the control of the pandemic. This review provided a comprehensive overview of the evidence for TCM treatments in severe/critical patients with COVID-19 as of 19 March 2022, including 21 studies enrolling 12,981 patients. Considering potential clinical heterogeneity due to different TCM treatments included, we performed qualitative systematic analyses and illustrated the effect and safety based on the trend and distribution of effect estimates. Compared to usual supportive treatment, TCM treatments showed consistent direction that TCM treatments could substantially reduce the risk of mortality, rate of conversion to critical cases, and rate of mechanical ventilation, shorten length of hospital/ICU stay, time to the nucleic acid conversion, time to symptom resolution, improve laboratory indicators, inflammatory biomarkers, and hematological indicators. In addition, Qigong exercise and acupressure rehabilitation program during the treatment period could shorten the length of hospital stay and improve lung function and symptoms. Although evidence remains inadequate, it was found that the use of TCM did not cause more adverse reactions among severe/critical patients and no serious adverse events related to TCM were found.

We failed to perform subgroup analyses because of insufficient number of studies for each outcome. However, a cohort study showed that more benefit in reducing the risk of mortality was found in patients without prior medical history/comorbidities status. Another cohort study showed that the risk of mortality increased by 5.0% with every one year of age increase. In addition, although some benefits were from studies with high risk of bias, the potential advantages of TCM treatments have still been well-documented.

### Potential Mechanism

Studies on the mechanism of TCM have gradually emerged in recent years and strived to explain the mechanism clearly in scientific methods. TCM treatment of COVID-19 has a wide range of effects and multiple targets, which can regulate the internal environment, enhance immunity, control systemic inflammatory response, improve patient symptoms, prevent acute respiratory distress syndrome (ARDS), and reduce risk of mortality. Control the local and systemic inflammatory response and a potentially life-threatening inflammatory cytokine storm presumably could reduce the severity and mortality rate of COVID-19 (Dhama et al., 2020; Mehta et al., 2020). Severe/critical patients were prone to decrease LYM and increase CRP levels (Hillas et al., 2010; Chan et al., 2020; Xu Z. et al., 2020), which aggravated the condition and endangered patients’ life. Evidence from our review showed that TCM could restore LYM and CRP to a general level and reduce the rate of critical cases. Zhao et al. (2021) investigated the mechanisms of QFPD against that of COVID-19 from the levels of molecule, pathway, and network; after comprehensive network and pathway analysis, the study indicated that four compounds (baicalin, glycyrrhizic acid, hesperidin, and hyperoside) and seven targets (AKT1, TNF-α, IL-6, prostaglandin-endoperoxide synthase (PTGS) 2, heme oxygenase 1 (HMOX1), IL-10, and TP53) were key molecules related to QFPD’s effects, and 55 important targets, which regulated five functional modules corresponding to QFPD’s effects in immune regulation, anti-infection, anti-inflammation, and multi-organ protection, respectively, were identified. This was important for promoting body temperature recovery and improving lung imaging, preventing further deterioration and reducing mortality in COVID-19 patients (Zhao et al., 2020a; Chen J. et al., 2020; Wu et al., 2020; Xu T. F. et al., 2020; Yan et al., 2020; Yang et al., 2020). XBJ had advantages in reducing inflammatory response and ARDS based on five active ingredients (hydroxysafflor yellow A, paeoniflorin oxide, *Ligusticum striatum* DC., lactone I, and paeoniflorin) (Sun et al., 2010; Ma et al., 2020). The molecular docking results of XBJ showed that the following effective ingredients and target binding activities were the best: ethyl ferulate–glyceraldehyde 3-phosphate dehydrogenase (GAPDH), protocatechuic acid–albumin (ALB) Rutin–TNF, apigenin–epidermal growth factor receptor (EGFR), ethy ferulate–mitogen-activated protein kinase (MAPK) 1, benzoylpaeoniflorin–caspase-3 (CASP-3), cryptotanshinone–signal transducer and activator of transcription (STAT)3, rosmarinic acid–MAPK8, cryptotanshinone–PTGS 2, and salvianolic acid B–transcription factor AP-1 (JUN). These active ingredients could synergistically produce anti-inflammatory and immunomodulatory effects (Zheng et al., 2020). The main active ingredients in CS included bufalin, resibufogenin, and cinobufagin, which possessed strong antiviral effects (Qi et al., 2018; Zhan et al., 2020). The result of molecular docking indicated that bufalin had the highest binding efficiency to 3CL protease, transcription factor AP-1 (ACE2), RNA-dependent RNA polymerase (RdRp), and spike protein and potentially suggested that CS had a good therapeutic effect on COVID-19 (Xu et al., 2021). The top eight compounds in HSBD were quercetin, baicalein, kaempferol, beta-sitosterol, stigmasterol, isorhamnetin, naringenin, and formononetin. These compounds had a strong affinity for ACE2 protein and SARS-CoV-2 3CL protein and could modulate signaling pathways to be effective in the treatment of COVID-19 (Tao et al., 2020). Major chemical components of XYP were andrographolide sulfate A, andrographolide sulfate B, and andrographolide sulfate C (Zhan et al., 2012), and a pharmacological study demonstrated that XYP had antiviral, antipyretic, and anti-inflammatory effects (Zhuang et al., 2020). The network pharmacology results of RDN showed that active components such as quercetin and trans-caffeic acid could regulate cellular inflammatory factors through pathways (Jia et al., 2021). Top 10 chemical components of YDZF were rutin, sennoside A, hyperoside, 4-hydroxycinnamic acid, sinapic acid, rhein, wogonin, atractylenolide III, emodin, and aloe emodin, and results of a network pharmacological study indicated that it could exert anti-inflammatory and immune regulation through TNF, PI3K-Akt, hypoxia-inducible factor (HIF)-1, and the toll-like receptor signaling pathway (Ma, 2021). The main chemical components of SM included beta-sitosterol, stigmasterol, and ginsenoside rh2, which could dock with core targets, such as caspase, estrogen receptor 1, catalase, and nuclear factor-κB inhibitor α, and reduce the level of inflammatory cytokines (Chen Z. W. et al., 2021). Key targets of HJSS, such as IL-6, TNF, and catalase relieved the acute lung injury by deregulating the sphingolipid signaling pathway by managing HIF-1 and NOD-like receptors (Hu H. et al., 2021).

TCM could shorten the duration of SARS-CoV-2 RNA persistence time, inhibit SARS-CoV-2 replication in Vero E6 cells, reduce the mRNA level (Lyu et al., 2021) and negative rate of nucleic acid. The components of TCM played a role in relieving the symptoms of COVID-19. For example, Semen Armeniacae Amarum (xinren) in QFPD could relieve cough and asthma, and Gypsum Fibrosum (Shengshigao) and Glycyrrhizae Radix (Gancao) contained in HSBD and QFPD could reduce fever, relieve cough, and regulate the immune system.

### Strengths and Limitations

Our study had several strengths. First, to the best of our knowledge, this study is an up-to-date systematic review based on all available evidence to evaluate the efficacy and safety of TCM for severe/critical patients. Second, we strictly followed the back-to-back principle for literature screening, data extraction, and bias risk assessment, which ensured the quality of the study in the methodological aspect. Third, considering the insufficient number of RCT evidence, we also included observational studies to supplement the body of evidence and improve the persuasiveness of our review. Finally, the implementation and reporting of the research followed internationally recognized standards to ensure quality and improve research readability. In addition, we focused on patient-important outcomes, which came from the integration of the “*Therapeutics and COVID-19: living guideline”* issued by the WHO and the *“Core Outcome Set for Clinical Trials on Coronavirus Disease 2019.”*

However, there were some limitations in this review. First, although we conducted a systematical search, continuously update, screened studies from more than 200,000 records, and included studies from both RCTs and observational studies, only 21 studies were included finally. Second, considering the limited number of included studies and different TCM treatments included, we only performed qualitative analysis without meta-analysis. However, in order to summarize the effect direction and enhance the reliability of the review, we drew clustered bar charts and effect direction plot to visual present the results. Third, although we performed sensitivity analysis by restricting studies to be low risk of bias and relatively robust results were found, our findings might be influenced by small sample size and high risk of bias studies. In addition, current pharmacological studies focusing on the mechanism of TCM failed to explore the mechanism from the perspective of chemical components and components’ percentage, which hindered our further discussion of the potential mechanism of TCM in the treatment of COVID-19. Future research is necessary to address this issue.

### Implications

The clinical understanding and treatment of diseases in TCM was not only differential diagnosis of disease but also differentiation of symptoms and signs. However, TCM treatment focuses on the difference of “syndrome,” and could further understand the disease through syndrome differentiation. Evidence-based evidence had shown that a variety of TCM possessed advantages in the treatment of COVID-19, but each TCM had its own characteristics. According to the existing guidelines for the COVID-19 (CAIM, 2020; Wang and Huang, 2020; Li et al., 2021; NHC, 2022), patients with dyspnea require mechanical ventilation. For patients accompanied by unconsciousness, irritability, cold sweat, and cold limbs, it was inner blocking causing collapse. Based on this syndrome type, XYP and RDN should be used in case of fever, cough and uncomfortable expectoration. If there were palpitations, shortness of breath, chills, perspiration due to deficiency of both qi and yin, and pulse deficiency, given SM. HSBD should be used if there exist the following symptoms: fever, cough, yellow and sticky sputum, or blood in sputum, fatigue, dry mouth, bitter and sticky, nausea, loss of appetite, and poor stool. XYP, XBJ, and RND were effective for syndrome of flaring heat in qifen and yingfen, that is, when the following symptoms occur: upset and thirsty, shortness of breath, delirium, dizziness, blurring of vision, or rash, or hematemesis, bleeding, or limb convulsions. In addition to TCM recommended in the published guidelines, the characteristics of patients included in the studies have shown that GZ 3 also had certain advantages for patients with inner blocking causing collapse (Huang et al., 2021). CHJD was suitable for patients with heat toxicity in the lungs and intestines. FZJF could be used in patients diagnosed with deficiency of vital energy (Wang et al., 2021a). The main symptoms of patients treated with YDZF and MHLJ were fever and dry cough (Zhao et al., 2020b; Chen G. et al., 2020). Similarly, more than 95% patients using HJSS had coughs (Hu H. et al., 2021).

At a time when the COVID-19 pandemic prevails around the world, China has posted excellent performance in pandemic prevention and COVID-19 treatment, maintained a low infection rate and a high cure rate of COVID-19 (NATCM 2022a; NATCM 2022b) and entered the “dynamic zero-case” stage of precise prevention and control. Among various prevention and control measures, TCM, as a treasure of traditional Chinese culture, once again safeguarded the health of the public and made significant contribution to the battle against the pandemic. In this context, the unique advantages of TCM should be utilized and should be ready to take the center stage globally and ultimately benefit the people all around the world.

According to “WHO Expert Meeting on Evaluation of Traditional Chinese Medicine in the Treatment of COVID-19 (28 February– 2 March 2022)” (WHO, 2022a), there was insufficient evidence (one RCT) for the treatment of severe/critical patients with TCM, but the safety of TCM was comparable to that of conventional western medicine. We included six RCTs and 15 observational studies in this review, TCM presented substantial advantages (e.g., reduction in the risk of mortality and rate of conversion to critical cases, improvement of inflammatory) in treating severe/critical COVID-19 patients. At present, TCM has been applied in 196 countries and regions around the world, and 86 countries have signed cooperation agreements on TCM with China. A total of 17 TCM centers have been established abroad, a few TCM standards have been formulated, and several TCM cooperation bases have been established (Wang and Wang, 2016; Wang, 2018). Among the “Three Medicines and Three Prescriptions” recommended in the Diagnosis and Treatment Protocol for Novel Coronavirus Pneumonia issued by the National Health Commission (China), HSBD have received emergency use approval in the United Arab Emirates and Cambodia; XFBD has been approved for sale by the Natural and Non-prescription Health Products Directorate (Canada); In Pakistan, JHQG has completed a drug registration–oriented clinical trial, proving its clear efficacy in the treatment of COVID-19. From 2012 to 2020, LHQW has been approved for sale in Canada, Mozambique, Singapore, Thailand, Ecuador, Laos, Brazil, and other countries. In the aforementioned context, strengthening international exchanges and carrying out close international cooperation is conducive to conducting high-quality and large-scale research on TCM and promoting TCM to the world. However, the application of TCM overseas must also consider the wishes of the local people comprehensively and be carried out in accordance with laws and regulations.

In the clinical practice perspective, the use of TCM should be as early as possible. In the existing clinical studies, TCM has shown that the earlier the interventional treatment was used, the better the prognosis of the patient would be (Liang et al., 2020; Yong et al., 2020; Zhang J. et al., 2021). In this regard, clinicians should fully consider the characteristics of the patient’s disease, and use TCM in a reasonable and timely manner to maximize the therapeutic effect.

## Conclusion

Compared to usual supportive treatment in severe/critical patients, TCM could significantly reduce rate of conversion of critical cases and mechanical ventilation, shorten time of nucleic acid conversion and length of hospital stay, improve PaO_2_/FiO_2_ and laboratory indicators, accelerate symptom recovery, and ultimately reduce mortality. TCM has a safety profile that is comparable to that of usual supportive treatment alone. The roles of TCM against COVID-19 have been well-documented, which sets an example of using traditional medicine in preventing and treating COVID-19 worldwide. However, the present conclusions might be influenced due to small sample size and high risk of bias in the randomization process generated and unadjusted confounders. More high-quality international multicenter researches and mechanism studies are still needed to further corroborate the effectiveness and safety of TCM in the treatment of COVID-19.

## Data Availability

The original contributions presented in the study are included in the article/Supplementary Material; further inquiries can be directed to the corresponding authors.

## Glossary

ALT: Alanine transaminase
AST: Aspartate aminotransferase
CBM: Chinese Biomedical Literature Database
CENTRAL: Cochrane Central Register of Controlled Trials
COVID-19: Coronavirus disease 2019
CRP: C-reactive protein
CNKI: China national knowledge infrastructure
CHJD: Chaihu Jiedu granules
CS: Chansu injection
Cr: Creatinine
CK: Creatine kinase
ESR: Erythrocyte sedimentation rate
FZJF: Fuzheng Jiufei granules
GZ 3: Gengzi No.3 recipe
HJSS: Hejie Shenshi decoction
HSBD: Huashi Baidu granules
IL-6: Interleukin 6
IFN-γ: Interferon-γ
LYM: Lymphocyte
MHLJ: Mahuang Liu Jun Tang
NC: Neutrophil count
PCT: Procalcitonin
PLT: Platelet count
QFPD: Qingfei Paidu decoction
RCT: Randomized Controlled Trial
RDN: Reduning injection
SARS-CoV-2: Syndrome coronavirus virus 2
SH: Shenghuang granules
SM: Shenmai injection
TCM: Traditional Chinese medicine
TNF-α: Tumor necrosis factor-α
VIP: China Science and Technology Journal Database
WHO: World Health Organization
WBC: White blood cell
XBJ: Xuebijing injection
XYP: Xiyanping injecton
YDZF: Yidu-toxicity blocking lung (NATCM)

## References

[B1] Agarwal A. Rochwerg B. Lamontagne F. Siemieniuk R. A. Agoritsas T. Askie L. (2020). A Living WHO Guideline on Drugs for Covid-19. BMJ 370, m3379. 10.1136/bmj.m3379 32887691

[B2] CAIM (2020). Chinese Association of Integrative Medicine. Expert Consensus on the Prevention and Treatment of Novel Coronavirus Pneumonia with Integrated Traditional Chinese and Western Medicine. Chin J Integr Tradit West Med 40 (12), 1413–1423. 10.7661/j.cjim.20200909.040

[B3] Chan J. F. Yuan S. Kok K. H. To K. K. Chu H. Yang J. (2020). A Familial Cluster of Pneumonia Associated with the 2019 Novel Coronavirus Indicating Person-To-Person Transmission: a Study of a Family Cluster. Lancet 395 (10223), 514–523. 10.1016/S0140-6736(20)30154-9 31986261 PMC7159286

[B4] Chen G. Su W. Yang J. Luo D. Xia P. Jia W. (2020). Chinese Herbal Medicine Reduces Mortality in Patients with Severe and Critical Coronavirus Disease 2019: a Retrospective Cohort Study. Front. Med. 14 (6), 752–759. 10.1007/s11684-020-0813-6 32926320 PMC7488644

[B5] Chen J. Wang Y. K. Gao Y. Hu L. S. Yang J. W. Wang J. R. (2020). Protection against COVID-19 Injury by Qingfei Paidu Decoction *via* Anti-viral, Anti-inflammatory Activity and Metabolic Programming. Biomed. Pharmacother. 129, 110281. 10.1016/j.biopha.2020.110281 32554251 PMC7247521

[B6] Chen Z. W. Wang D. Y. Zhang B. J. Zhou J. Du H. (2021). Network Pharmacology Analysis and Clinical Verification of Shenmai Injection in the Treatment of Knee Osteoarthritis. Chin. J. Bone Jt. 10 (07), 546–553. 10.3969/j.issn.2095-252X.2021.07.012

[B7] Chen. L. Zhang A. Li Q. T. Cui Y. Yuan G. D. (2021). Evaluation of Clinical Value of Xuebijing Combined with Human Immunoglobulin in Severe and Critically Ill Patients with Coronavirus Disease 2019. Chin. Crit. Care Med. 33 (04), 399–404. 10.3760/cma.j.cn121430-20200628-00490 34053480

[B8] Dhama K. Patel S. K. Pathak M. Yatoo M. I. Tiwari R. Malik Y. S. (2020). An Update on SARS-CoV-2/covid-19 with Particular Reference to its Clinical Pathology, Pathogenesis, Immunopathology and Mitigation Strategies. Travel Med. Infect. Dis. 37, 101755. 10.1016/j.tmaid.2020.101755 32479816 PMC7260597

[B9] Fan T. T. Cheng B. L. Fang X. M. Chen Y. C. Su F. (2020). Application of Chinese Medicine in the Management of Critical Conditions: A Review on Sepsis. Am. J. Chin. Med. 48 (6), 1315–1330. 10.1142/S0192415X20500640 32907362

[B10] Feng J. Fang B. Zhou D. Wang J. Zou D. Yu G. (2021). Clinical Effect of Traditional Chinese Medicine Shenhuang Granule in Critically Ill Patients with COVID-19: A Single-Centered, Retrospective, Observational Study. J. Microbiol. Biotechnol. 31 (3), 380–386. 10.4014/jmb.2009.09029 33746189 PMC9705840

[B11] Ge L. Zhu H. Wang Q. Li M. Cai J. Chen Y. (2021). Integrating Chinese and Western Medicine for COVID-19: A Living Evidence-Based Guideline (Version 1). J. Evid. based Med. 14 (4), 313–332. 10.1111/jebm.12444 34632732

[B12] Hillas G. Vassilakopoulos T. Plantza P. Rasidakis A. Bakakos P. (2010). C-reactive Protein and Procalcitonin as Predictors of Survival and Septic Shock in Ventilator-Associated Pneumonia. Eur. Respir. J. 35 (4), 805–811. 10.1183/09031936.00051309 19717486

[B13] Hu F. Chen J. Chen H. Zhu J. Wang C. Ni H. (2021). Chansu Improves the Respiratory Function of Severe COVID-19 Patients. Pharmacol. Res. - Mod. Chin. Med. 1, 100007. 10.1016/j.prmcm.2021.100007

[B14] Hu H. Wang K. Wang L. Du Y. Chen J. Li Y. (2021). He-Jie-Shen-Shi Decoction as an Adjuvant Therapy on Severe Coronavirus Disease 2019: A Retrospective Cohort and Potential Mechanistic Study. Front. Pharmacol. 12, 700498. 10.3389/fphar.2021.700498 34220524 PMC8250425

[B15] Huang D. H. Yuan Q. L. Tao C. H. Xia G. W. An C. Q. Fan C. Y. (2021). Effect of Gengzi No.3 Recipe on Neutrophils to Lymphocyte Ratio of Inner Blocking Causing Collapse Syndrome Caused by Severe COVID-19. Shanxi TCM 37 (7), 11–14. 10.3969/j.issn.1000-7156.2021.07.006

[B16] Jia S. Luo H. Liu X. Fan X. Huang Z. Lu S. (2021). Dissecting the Novel Mechanism of Reduning Injection in Treating Coronavirus Disease 2019 (COVID-19) Based on Network Pharmacology and Experimental Verification. J. Ethnopharmacol. 273, 113871. 10.1016/j.jep.2021.113871 33485971 PMC7825842

[B17] Jin X. Pang B. Zhang J. Liu Q. Yang Z. Feng J. (2020). Core Outcome Set for Clinical Trials on Coronavirus Disease 2019 (COS-COVID). Eng. (Beijing) 6 (10), 1147–1152. 10.1016/j.eng.2020.03.002 PMC7102592 32292626

[B18] Li Z. Y. Xie Z. J. Li H. C. Wang J. J. Wen X. H. Wu S. Y. (2021). Guidelines on the Treatment with Integrated Traditional Chinese Medicine and Western Medicine for Severe Coronavirus Disease 2019. Pharmacol. Res. 174, 105955. 10.1016/j.phrs.2021.105955 34715330 PMC8553423

[B19] Liang L. X. Pan H. D. Huang Y. F. (2020). The Scientific Basis of Traditional Chinese Medicine in the Treatment of Novel Coronavirus Pneumonia. Engineering 6 (10), 94–112. 10.1016/j.eng.2020.08.009

[B20] Liu S. T. Zhan C. Ma Y. J. Guo C. Y. Chen W. Fang X. M. (2021). Effect of Qigong Exercise and Acupressure Rehabilitation Program on Pulmonary Function and Respiratory Symptoms in Patients Hospitalized with Severe COVID-19: a Randomized Controlled Trial. Integr. Med. Res. 10, 100796. 10.1016/j.imr.2021.100796 34733607 PMC8553411

[B21] Liu X. S. Song Y. L. Guan W. J. Qiu H. B. Du B. Li Y. M. (2021). A Multicenter Prospective Cohort Study of Xuebijing Injection in the Treatment of Severe Coronavirus Disease 2019. Chin. Crit. Care Med. 33 (07), 774–778. 10.3760/cma.j.cn121430-20210514-00714 34412743

[B22] Liu Y. J. (2021). Analysis of Clinical Efficacy of Western Medicine Combined with Huashibaidu Decoction in the Treatment of COVID-19. Lab. Med. Clin. 18 (08), 1152–1153. 10.3969/j.issn.1672-9455.2021.08.037

[B23] Luo Z. Chen W. Xiang M. Wang H. Xiao W. Xu C. (2021). The Preventive Effect of Xuebijing Injection against Cytokine Storm for Severe Patients with COVID-19: A Prospective Randomized Controlled Trial. Eur. J. Integr. Med. 42, 101305. 10.1016/j.eujim.2021.101305 33552315 PMC7847185

[B24] Lyu M. Fan G. Xiao G. Wang T. Xu D. Gao J. (2021). Traditional Chinese Medicine in COVID-19. Acta Pharm. Sin. B 11 (11), 3337–3363. 10.1016/j.apsb.2021.09.008 34567957 PMC8450055

[B25] Ma Q. Qiu M. Zhou H. Chen J. Yang X. Deng Z. (2020). The Study on the Treatment of Xuebijing Injection (XBJ) in Adults with Severe or Critical Corona Virus Disease 2019 and the Inhibitory Effect of XBJ against SARS-CoV-2. Pharmacol. Res. 160, 105073. 10.1016/j.phrs.2020.105073 32653650 PMC7347326

[B26] Ma Y. (2021). Pharmacodynamic Material Basis and Molecular Mechanism of Yingdu Shufei Recipe in the Treatment of New Coronary Pneumonia. Yichun College.

[B27] MAGIC (2022a). How to Rate Risk of Bias in Observational Studies. [Online]. Available at: http://help.magicapp.org/knowledgebase/articles/294933-how-to-rate-risk-of-bias-in-observational-studies (Accessed March 20, 2022).

[B28] MAGIC (2022b). How to Rate Risk of Bias in Randomized Controlled Trials. [Online]. Available at: http://help.magicapp.org/knowledgebase/articles/294932-how-to-rate-risk-of-bias-in-randomized-controlled (Accessed March 20, 2022).

[B29] Mehta P. McAuley D. F. Brown M. Sanchez E. Tattersall R. S. Manson J. J. (2020). COVID-19: Consider Cytokine Storm Syndromes and Immunosuppression. Lancet 395 (10229), 1033–1034. 10.1016/S0140-6736(20)30628-0 32192578 PMC7270045

[B30] NATCM National Administration of Traditional Chinese Medicine (2022a). Experience of Traditional Chinese Medicine in Treating COVID-19. Avaliable from: http://www.satcm.gov.cn/xinxifabu/meitibaodao/2020-04-01/14418.html (Accessed April 2, 2022).

[B31] NATCM National Administration of Traditional Chinese Medicine (2022b). Two 90% Tell You How Strong Chinese Medicine Is in the Treatment of COVID-19. Avaliable from: http://www.satcm.gov.cn/xinxifabu/meitibaodao/2020-04-07/14511.html (Accessed April 2, 2022).

[B32] NHC (2022). The NHC of the People’s Republic of China. The Ninth Edition Guideline for Diagnosis and Treatment COVID-19. The National Health Commission of the People’s Republic of China. [Online]. Available: http://www.gov.cn/zhengce/zhengceku/2022-03/15/content_5679257.htm (Accessed April 2, 2022)

[B33] Ouzzani M. Hammady H. Fedorowicz Z. Elmagarmid A. (2016). Rayyan-a Web and Mobile App for Systematic Reviews. Syst. Rev. 5 (1), 210. 10.1186/s13643-016-0384-4 27919275 PMC5139140

[B34] Page M. J. McKenzie J. E. Bossuyt P. M. Boutron I. Hoffmann T. C. Mulrow C. D. (2021). The PRISMA 2020 Statement: an Updated Guideline for Reporting Systematic Reviews. Bmj 372, n71. 10.1136/bmj.n71 33782057 PMC8005924

[B35] Qi J. Zulfiker A. H. M. Li C. Good D. Wei M. Q. (2018). The Development of Toad Toxins as Potential Therapeutic Agents. Toxins (Basel) 10 (8), 336. 10.3390/toxins10080336 PMC6115759 30127299

[B36] Qin Y. L. Song Y. Y. Zhou L. Cui Y. Zhang A. (2020). Clinical Efficacy of Reduning Injection Combined with Methylprednisolone in the Treatment of Severe Coronavirus Disease 2019. Chin. Phram 29 (09), 19–22.

[B37] Runfeng L. Yunlong H. Jicheng H. Weiqi P. Qinhai M. Yongxia S. (2020). Lianhuaqingwen Exerts Anti-viral and Anti-inflammatory Activity against Novel Coronavirus (SARS-CoV-2). Pharmacol. Res. 156, 104761. 10.1016/j.phrs.2020.104761 32205232 PMC7102548

[B38] Shu Z. Chang K. Zhou Y. Peng C. Li X. Cai W. (2021). Add-On Chinese Medicine for Coronavirus Disease 2019 (ACCORD): A Retrospective Cohort Study of Hospital Registries. Am. J. Chin. Med. 49 (3), 543–575. 10.1142/S0192415X21500257 33683189

[B39] Siemieniuk R. A. Bartoszko J. J. Ge L. Zeraatkar D. Izcovich A. Kum E. (2020). Drug Treatments for Covid-19: Living Systematic Review and Network Meta-Analysis. BMJ 370, m2980. 10.1136/bmj.m2980 32732190 PMC7390912

[B40] Sterne J. A. C. Savović J. Page M. J. Elbers R. G. Blencowe N. S. Boutron I. (2019). RoB 2: a Revised Tool for Assessing Risk of Bias in Randomized Trials. BMJ 366, l4898. 10.1136/bmj.l4898 31462531

[B41] Sterne J. A. Hernán M. A. Reeves B. C. Savović J. Berkman N. D. Viswanathan M. (2016). ROBINS-I: a Tool for Assessing Risk of Bias in Non-randomised Studies of Interventions. BMJ 355, i4919. 10.1136/bmj.i4919 27733354 PMC5062054

[B42] Sun J. Xue Q. Guo L. Cui L. Wang J. (2010). Xuebijing Protects against Lipopolysaccharide-Induced Lung Injury in Rabbits. Exp. Lung Res. 36 (4), 211–218. 10.3109/01902140903312123 20426529

[B43] Sun Q. G. An X. D. Xie P. Jiang B. Tian J. X. Yang Q. (2021). Traditional Chinese Medicine Decoctions Significantly Reduce the Mortality in Severe and Critically Ill Patients with COVID-19: A Retrospective Cohort Study. Am. J. Chin. Med. 49 (5), 1063–1092. 10.1142/S0192415X21500518 34107858

[B44] Tao Q. Du J. Li X. Zeng J. Tan B. Xu J. (2020). Network Pharmacology and Molecular Docking Analysis on Molecular Targets and Mechanisms of Huashi Baidu Formula in the Treatment of COVID-19. Drug Dev. Ind. Pharm. 46 (8), 1345–1353. 10.1080/03639045.2020.1788070 32643448 PMC7441778

[B45] Wang G. Q. (2018). Director of the State Administration of Traditional Chinese Medicine: Traditional Chinese Medicine Still Has a Long Way to Go. Available at: http://www.gov.cn/govweb/jrzg/2012-04/26/content_2124205.htm (Accessed April 10, 2022).

[B46] Wang G. Q. Wang J. P. (2016). Traditional Chinese Medicine, Going Out with Dignity. Available at: http://www.cntcm.com.cn/2016-03/02/content_12057.htm (Accessed April 10, 2022).

[B47] Wang Q. Li N. Li J. He Y. Li Y. Zhong D. (2021). A Protocol of a Guideline to Establish the Evidence Ecosystem of Acupuncture. Front. Med. 8, 711197. 10.3389/fmed.2021.711197 PMC8896352 35252220

[B48] Wang Y. Liu Y. Lv Q. Zheng D. Zhou L. Ouyang W. (2021a). Effect and Safety of Chinese Herbal Medicine Granules in Patients with Severe Coronavirus Disease 2019 in Wuhan, China: a Retrospective, Single-Center Study with Propensity Score Matching. Phytomedicine 85, 153404. 10.1016/j.phymed.2020.153404 33637412 PMC7642753

[B49] Wang Y. Lu C. Li H. Qi W. Ruan L. Bian Y. (2021b). Efficacy and Safety Assessment of Severe COVID-19 Patients with Chinese Medicine: A Retrospective Case Series Study at Early Stage of the COVID-19 Epidemic in Wuhan, China. J. Ethnopharmacol. 277, 113888. 10.1016/j.jep.2021.113888 33529638 PMC7847283

[B50] Wang Y. Y. Huang L. Q. (2020). Novel Coronavirus Pneumonia Prevention and Control Research Group of Zhongnan Hospital of Wuhan University. A Clinical Diagnosis & Treatment Rapid Advice Guideline for Integrating Chinese and Western Medicine of COVID-19. Chine Res. Hosp. 7 (02), 51–64. 10.1186/s40779-020-00245-9

[B51] Wen L. Zhou Z. Jiang D. Huang K. (2020). Effect of Xuebijing Injection on Inflammatory Markers and Disease Outcome of Coronavirus Disease 2019. Zhonghua Wei Zhong Bing Ji Jiu Yi Xue 32 (04), 426–429. 10.3760/cma.j.cn121430-20200406-00386 32527346

[B52] WHO (2020). Novel Coronavirus – China. [Online]. Available: http://www.who.int/csr/don/12-january-2020-novel-coronavirus-china/en/ (Accessed March 10, 2022).

[B53] WHO (2022b). Weekly Epidemiological Update on COVID-19. [Online]. Available: https://www.who.int/publications/m/item/weekly-epidemiological-update-on-covid-19---8-march-2022 (Accessed March 10, 2022).

[B54] WHO (2022a). WHO Expert Meeting on Evaluation of Traditional Chinese Medicine in the Treatment of COVID-19. [Online]. Available: https://www.who.int/publications/m/item/who-expert-meeting-on-evaluation-of-traditional-chinese-medicine-in-the-treatment-of-covid-19 (Accessed March 10, 2022).

[B55] Wu H. Wang J. Q. Yang Y. W. Li T. Y. Cao Y. J. Qu Y. X. (2020). Preliminary Exploration of the Mechanism of Qingfei Paidu Decoction against Novel Coronavirus Pneumonia Based on Network Pharmacology and Molecular Docking Technology. Acta Pharm. 55, 374–383. 10.16438/j.0513-4870.2020-0136

[B56] Xiong Y. Tian Y. Ma Y. Liu B. Ruan L. Lu C. (2022). The Effect of Huashibaidu Formula on the Blood Oxygen Saturation Status of Severe COVID-19: A Retrospective Cohort Study. Phytomedicine 95, 153868. 10.1016/j.phymed.2021.153868 34929564 PMC8641428

[B57] Xu T. F. He C. G. Yang H. (2020). Network Pharmacology-Based Study on Material Basis and Mechanism of Qingfei Paidu Decoction against Novel Coronavirus Pneumonia. Nat. Prod. Res. Dev. 32 (06), 901–908. 10.16333/j.1001-6880.2020.6.001

[B58] Xu Y. Peng W. Han D. Wang Z. Feng F. Zhou X. (2021). Material Basis and Mechanism of Chansu Injection for COVID-19 Treatment Based on Network Pharmacology and Molecular Docking Technology. Evidence-Based Complementary Altern. Med. 2021, 11. 10.1155/2021/7697785 PMC8523246 34671410

[B59] Xu Z. Shi L. Wang Y. Zhang J. Huang L. Zhang C. (2020). Pathological Findings of COVID-19 Associated with Acute Respiratory Distress Syndrome. Lancet Respir. Med. 8 (4), 420–422. 10.1016/S2213-2600(20)30076-X 32085846 PMC7164771

[B60] Yan H. Zou Y. Zou C. (2020). Mechanism of Qingfei Paidu Decoction for Treatment of COVID-19: Analysis Based on Network Pharmacology and Molecular Docking Technology. Nan Fang. Yi Ke Da Xue Xue Bao 40 (5), 616–623. 10.12122/j.issn.1673-4254.2020.05.02 32897211 PMC7277311

[B61] Yang R. Liu H. Bai C. Wang Y. Zhang X. Guo R. (2020). Chemical Composition and Pharmacological Mechanism of Qingfei Paidu Decoction and Ma Xing Shi Gan Decoction against Coronavirus Disease 2019 (COVID-19): In Silico and Experimental Study. Pharmacol. Res. 157, 104820. 10.1016/j.phrs.2020.104820 32360484 PMC7194979

[B62] Yong W. X. Feng C. Q. Zhang L. Y. (2020). Four Cases of Integrated Traditional Chinese and Western Medicine Treatment of Novel Coronavirus Pneumonia in Gansu. Shanghai J. Tradit. Chin. Med. 54 (03), 21–24. 10.16305/j.1007-1334.2020.03.006

[B63] Zhan H. Z. Chen W. K. Xiao X. W. Yang X. L. Luo Y. H. (2012). HPLC Simultaneous Determination of Four Effective Ingredients in Xiyanping Injection. Chin. J. Pharm. Anal. 32, 140–143. 10.16155/j.0254-1793.2012.01.015

[B64] Zhan X. Wu H. Wu H. Wang R. Luo C. Gao B. (2020). Metabolites from *Bufo gargarizans* (Cantor, 1842): A Review of Traditional Uses, Pharmacological Activity, Toxicity and Quality Control. J. Ethnopharmacol. 246, 112178. 10.1016/j.jep.2019.112178 31445132

[B65] Zhang J. Shu T. T. Luo W. D. (2021). A Retrospective Clinical Study on the Effect of Early Intervention of Traditional Chinese Medicine on the Disease Outcome of Severe Patients with Novel Coronavirus Pneumonia. JTradit Chin. Med. 62 (12), 1046–1051.

[B66] Zhang L. Zheng X. Bai X. Wang Q. Chen B. Wang H. (2021). Association between Use of Qingfei Paidu Tang and Mortality in Hospitalized Patients with COVID-19: A National Retrospective Registry Study. Phytomedicine 85, 153531. 10.1016/j.phymed.2021.153531 33799224 PMC7914374

[B67] Zhang P. Pan G. T. (2021). Clinical Study of Qingfei Paidu Decoction in Improving Inflammatory Cytokines in Critical Patients with Novel Coronavirus Pneumonia. World Sci. Technology-Modernization Traditional Chin. Med. 23 (02), 391–395. 10.11842/wst.20200416003

[B68] Zhao J. Tian S. Lu D. Yang J. Zeng H. Zhang F. (2021). Systems Pharmacological Study Illustrates the Immune Regulation, Anti-infection, Anti-inflammation, and Multi-Organ Protection Mechanism of Qing-Fei-Pai-Du Decoction in the Treatment of COVID-19. Phytomedicine 85, 153315. 10.1016/j.phymed.2020.153315 32978039 PMC7480398

[B69] Zhao J. Yang X. Wang C. Song S. Cao K. Wei T. (2020b). Yidu-toxicity Blocking Lung Decoction Ameliorates Inflammation in Severe Pneumonia of SARS-COV-2 Patients with Yidu-Toxicity Blocking Lung Syndrome by Eliminating IL-6 and TNF-A. Biomed. Pharmacother. 129, 110436. 10.1016/j.biopha.2020.110436 32768938 PMC7303599

[B70] Zhao J. Tian S. S. Yang J. Liu J. F. Zhang W. D. (2020a). Investigating Mechanism of Qing-Fei-Pai-Du-Tang for Treatment of COVID-19 by Network Pharmacology. Chin. Trad. Herb. Drugs 4 (51), 829–835. 10.7501/j.issn.0253-2670.2020.04.001

[B71] Zheng W. J. Yan Q. Ni Y. S. Zhan S. F. Yang L. L. Zhuang H. F. (2020). Examining the Effector Mechanisms of Xuebijing Injection on COVID-19 Based on Network Pharmacology. BioData Min. 13, 17. 10.1186/s13040-020-00227-6 33082858 PMC7563914

[B72] Zhou S. Feng J. Xie Q. Huang T. Xu X. Zhou D. (2021). Traditional Chinese Medicine Shenhuang Granule in Patients with Severe/critical COVID-19: A Randomized Controlled Multicenter Trial. Phytomedicine 89, 153612. 10.1016/j.phymed.2021.153612 34126419 PMC8161732

[B73] Zhuang W. Fan Z. Chu Y. Wang H. Yang Y. Wu L. (2020). Chinese Patent Medicines in the Treatment of Coronavirus Disease 2019 (COVID-19) in China. Front. Pharmacol. 11, 1066. 10.3389/fphar.2020.01066 32848729 PMC7396557

