# Supplementary material for: Role of Traditional Chinese Medicine in Treating Severe or Critical COVID-19: A Systematic Review of Randomized Controlled Trials and Observational Studies
Source: Front Pharmacol. 2022 Jul 15;13:926189. doi: 10.3389/fphar.2022.926189 (PMC9336221; doi:10.3389/fphar.2022.926189)
Supplement: Supplementary file 1 [file DataSheet1.docx]

**Role of Traditional Chinese Medicine for Treating Severe/Critical COVID-19: A Systematic Review of Randomized Trials and Observational Studies**

Mengting Li^1,2✝^, Hongfei Zhu^1,2^^✝^, Yafei Liu^1,2^, Yao Lu^1,2^, Minyao Sun^3^, Yuqing Zhang^4,5,6,7^, Jiaheng Shi^8,9^, Nannan Shi^8^, Ling Li^10^, Kehu Yang^11,12,13^, Xin Sun^10*^, Jie Liu^8,14*^, Long Ge^1,2,12,13*^, Luqi Huang^8,15*^

1 Department of Social Medicine and Health Management, School of Public Health, Lanzhou University, Lanzhou, China

2 Evidence Based Social Science Research Centre, School of Public Health, Lanzhou University, Lanzhou, China

3 Evidence Based Nursing Centre, School of Nursing, Lanzhou University, Lanzhou, China

4 Department of Health Research Methods, Evidence, and Impact, McMaster University, Hamilton, ON, Canada

5 CEBIM (Center for Evidence Based Integrative Medicine)-Clarity Collaboration, Guang’ anmen Hospital, China Academy of Chinese Medical Sciences, Beijing, China

6 Institute of Acupuncture and Moxibustion, China Academy of Chinese Medical Sciences, Beijing, China

7 Nottingham Ningbo GRADE Center, The University of Nottingham Ningbo, China

8 China Center for Evidence Based Traditional Chinese Medicine, China Academy of Chinese Medical Sciences, Beijing, China

9 Department of Emergency, Guang’ anmen Hospital, China Academy of Chinese Medical Sciences, Beijing, China

10 Chinese Evidence-Based Medicine Center, West China Hospital, Sichuan University, Chengdu, China

11 Evidence-Based Medicine Center, School of Basic Medical Sciences, Lanzhou University, Lanzhou, China

12 WHO Collaborating Center for Guideline Implementation and Knowledge Translation, Lanzhou, China

13 Key Laboratory of Evidence Based Medicine and Knowledge Translation of Gansu Province, Lanzhou, China

14 Department of Oncology, Guang’ anmen Hospital, China Academy of Chinese Medical Sciences, Beijing, China

15 National Resource Center for Chinese Materia Medica, China Academy of Chinese Medical Sciences, Beijing 100700, China.

✝ These authors have contributed equally to this work and share first authorship.

*Co-corresponding authors

Xinsun, Professor, Chinese Evidence-Based Medicine Center, West China Hospital, Sichuan University, Chengdu, China. E-mail address: sunxin@wchscu.cn

Jie Liu, Professor, China Center for Evidence Based Traditional Chinese Medicine, China Academy of Chinese Medical Sciences, Beijing, China. E-mail address: dr.liujie@163.com

Luqi Huang, Professor, China Center for Evidence Based Traditional Chinese Medicine/ National Resource Center for Chinese Materia Medica, China Academy of Chinese Medical Sciences, Beijing, China. E-mail address: huangluqi01@126.com

Long Ge, Researcher, Evidence Based Social Science Research Centre, School of Public Health, Lanzhou University. E-mail address: gelong2009@163.com

[**Table S1 Detailed search strategy** 4](#_Toc104453623)

[**Table S2 Detailed guidance for assessment of risk of bias** 8](#_Toc104453624)

[**Table S3 Characteristics of included studies** 12](#_Toc104453625)

[**Table S4 Studies reporting treatment of COVID-19 patients** 16](#_Toc104453626)

[**Table S5 Risk of bias of included RCTs** 22](#_Toc104453627)

[**Table S6 Risk of bias of included observational studies** 23](#_Toc104453628)

[**Table S7 Summary of findings on mortality** 24](#_Toc104453629)

[**Table S8 Summary of results of rate/time of mechanical ventilation** 26](#_Toc104453630)

[**Table S9 Summary of results of rate/time of the nucleic acid conversion** 27](#_Toc104453631)

[**Table S10 Summary of results of rate of conversion to moderate/critical cases** 28](#_Toc104453632)

[**Table S11 Summary of results of rate/time of symptom resolution** 29](#_Toc104453633)

[**Table S12 Summary of results of discharge rate and length of hospital stay** 30](#_Toc104453634)

[**Table S13 Summary of results of laboratory markers** 31](#_Toc104453635)

[**Table S14 Summary of results of PaO2/FiO2** 41](#_Toc104453636)

[**Table S15 Incidence of adverse reactions** 42](#_Toc104453637)

**Table S1 Detailed search strategy**

| **Databases [Platform]** | **Results** |
| --- | --- |
| CNKI | 1531 |
| WANFANG | 750 |
| CBM | 10,942 |
| VIP | 3,173 |
| PubMed | 72,746 |
| Cochrane Library | 8,901 |
| Embase | 48,654 |
| Web of Science | 65,572 |
| LOVE | 3,492 |
| **subotal** | **215,761** |
| **Duplicate** | **98,970** |
| **Total** | **116,791** |

**CNKI** March 19, 2022

Searched a sub-set of CNKI named *Open Access Online-First Publication Knowledge Service Platform of Fighting against Novel Coronavirus Pneumonia*, which is a special database collected COVID-19 literatures. Therefor, we only searched study design in this database.

Search Strategy:

| **#** | **Searches** | **Results** |
| --- | --- | --- |
| 1 | SU = (“随机” + “对照” + “队列” + “观察” + “观察” + “试验” + “临床”+ “临床回顾” + “病例对照” + “多中心回顾性研究”) | 542 |
| 2 | TKA = (“随机” + “对照” + “队列” + “观察” + “观察” + “试验” + “临床”+ “临床回顾” + “病例对照” + “多中心回顾性研究”) | 1,531 |
| 3 | 1 OR 2 AND Date:2019-* | 1,531 |

**WANFANG** March 19, 2022

Search Strategy:

| **#** | **Searches** | **Results** |
| --- | --- | --- |
| 1 | 题名或关键词:(COVID-19 or COVID19 or COVID-2019 or SARS-CoV-19 or SARS-CoV-2019 or SARS-CoV-2 or SARS2 or 2019-nCoV or 严重急性呼吸综合征冠状病毒2型 or 冠状病毒感染or 武汉冠状病毒 or 武汉海鲜市场肺炎病毒or 冠状病毒2019 or SARS-CoV-19 or SARS-CoV-2019 or 2019新型冠状病毒 or 2019新型冠状病毒感染 or 2019冠状病毒 or 新型冠状病毒-19 or 冠状病毒) | 80,594 |
| 2 | 题名或关键词:(随机 or 对照 or 队列 or 观察 or 疗效 or 试验 or 临床or 临床回顾 or 病例对照 or 多中心回顾性研究) | 6,557,937 |
| 3 | 1 AND 2 AND Date:2019-* | 750 |

**CBM** March 19, 2022

Search Strategy:

| **#** | **Searches** | **Results** |
| --- | --- | --- |
| 1 | "SARS病毒"[不加权:扩展] | 7055 |
| 2 | "COVID-19"[常用字段:智能] OR "COVID19"[常用字段:智能] OR "COVID-2019"[常用字段:智能] OR "SARS-CoV-19"[常用字段:智能] OR "SARS-CoV-2019"[常用字段:智能] OR "SARS-CoV-2"[常用字段:智能] OR "SARS2"[常用字段:智能] OR "2019-nCoV"[常用字段:智能] OR "严重急性呼吸综合征冠状病毒2型"[常用字段:智能] OR "冠状病毒感染"[常用字段:智能] OR "武汉冠状病毒"[常用字段:智能] OR "武汉海鲜市场肺炎病毒"[常用字段:智能] OR "冠状病毒2019"[常用字段:智能] OR "2019新型冠状病毒"[常用字段:智能] OR "2019新型冠状病毒感染"[常用字段:智能] OR "2019冠状病毒"[常用字段:智能] OR "新型冠状病毒-19"[常用字段:智能] OR "冠状病毒"[常用字段:智能] | 35,405 |
| 3 | 1 OR 2 | 35,714 |
| 4 | "随机对照试验"[不加权:扩展] | 499,796 |
| 5 | "病例对照研究"[不加权:扩展] | 1,611,709 |
| 6 | "随机"[常用字段:智能] OR "对照"[常用字段:智能] OR "队列"[常用字段:智能] OR "观察"[常用字段:智能] OR "疗效"[常用字段:智能] OR "试验"[常用字段:智能] OR "临床"[常用字段:智能] OR "疗效"[常用字段:智能] OR "临床回顾"[常用字段:智能] OR "病例对照"[常用字段:智能] OR "多中心回顾性研究"[常用字段:智能] | 6,595,253 |
| 7 | 4 OR 5 OR 6 | 6,675,946 |
| 8 | 3 AND 7 AND 2019-2022[日期] | 10,942 |

VIP March 19, 2022

| 1 | M=("COVID-19" OR "COVID19" OR "COVID-2019" OR "SARS-CoV-19" OR "SARS-CoV-2019" OR "SARS-CoV-2" OR "SARS2" OR "S2019-nCoV" OR "严重急性呼吸综合征冠状病毒2型" OR "冠状病毒感染" OR "武汉冠状病毒" OR "武汉海鲜市场肺炎病毒" OR "冠状病毒2019" OR "新型冠状病毒" OR "2019新型冠状病毒感染" OR "2019冠状病毒" OR "新型冠状病毒-19" OR "冠状病毒") | 34,418 |
| --- | --- | --- |
| 2 | M=("随机" OR “对照”OR "队列" OR "观察" OR "疗效" OR "试验" OR 临床 OR "临床回顾" OR "多中心回顾性研究") | 3,838,482 |
| 3 | 1 AND 2 AND (years: [2019 TO 2022]) | 3,173 |

**PubMed** 2019/01/01-2021/3/19

Search Strategy:

| **#** | **Searches** | **Results** |
| --- | --- | --- |
| 1 | "COVID-19"[Mesh] | 146,853 |
| 2 | "SARS-CoV-2"[Mesh] | 117,877 |
| 3 | coronavirus[Title/Abstract] OR "corona virus"[Title/Abstract] OR coronavirinae[Title/Abstract] OR coronaviridae[Title/Abstract] OR betacoronavirus[Title/Abstract] OR covid19[Title/Abstract] OR "covid 19"[Title/Abstract] OR nCoV[Title/Abstract] OR "CoV 2"[Title/Abstract] OR CoV2[Title/Abstract] OR sarscov2[Title/Abstract] OR 2019nCoV[Title/Abstract] OR "novel CoV"[Title/Abstract] OR "wuhan virus"[Title/Abstract] OR "COVID-19"[Title/Abstract] OR "COVID-2019"[Title/Abstract] OR "SARS-CoV-19"[Title/Abstract] OR "SARS-CoV-2019"[Title/Abstract] OR "SARS-CoV-2"[Title/Abstract] OR "SARS2"[Title/Abstract] OR "2019-nCoV"[Title/Abstract] | 240,408 |
| 4 | 1-3/OR | 246,984 |
| 5 | "Randomized Controlled Trial" [Publication Type] | 563,075 |
| 6 | "Randomized Controlled Trials as Topic"[Mesh] | 157,351 |
| 7 | "Case-Control Studies"[Mesh] | 1,296,884 |
| 8 | "Cohort Studies"[Mesh] | 2,313,454 |
| 9 | "Controlled Before-After Studies"[Mesh] | 690 |
| 10 | "Cross-Sectional Studies"[Mesh] | 415,829 |
| 11 | "Historically Controlled Study"[Mesh] | 220 |
| 12 | "randomized controlled trial"[Title/Abstract] OR "controlled clinical trial"[Title/Abstract] OR "randomized"[Title/Abstract] OR "placebo"[Title/Abstract] OR "randomly"[Title/Abstract] OR "trial"[Title/Abstract] OR "clinical research"[Title/Abstract] OR "clinical observation"[Title/Abstract] OR "control"[Title/Abstract] OR cohort*[Title/Abstract] OR prospective[Title/Abstract] OR longitudinal[Title/Abstract] OR "follow up"[Title/Abstract] OR "case control"[Title/Abstract] OR "case referent"[Title/Abstract] OR "case stud*"[Title/Abstract] OR "case series"[Title/Abstract] OR "cross sectional"[Title/Abstract] | 6,133,486 |
| 13 | 5-12/OR | 7,346,068 |
| 14 | 4 AND 13 | 74,472 |
| 15 | 14 AND ("2019/01/01"[Date - Create] : "2022/3/19"[Date - Create]) | 72,746 |

**Embase** 2019/01/01-2022/03/19

Search Strategy:

| **#** | **Searches** | **Results** |
| --- | --- | --- |
| 1 | 'coronavirus disease 2019'/exp | 197,781 |
| 2 | coronavirus:ab,ti OR 'corona virus':ab,ti OR coronavirinae:ab,ti OR coronaviridae:ab,ti OR betacoronavirus:ab,ti OR covid19:ab,ti OR 'covid 19':ab,ti OR nCoV:ab,ti OR 'CoV 2':ab,ti OR CoV2:ab,ti OR sarscov2:ab,ti OR 2019nCoV:ab,ti OR 'novel CoV':ab,ti OR 'wuhan virus':ab,ti OR 'COVID-19':ab,ti OR 'COVID-2019':ab,ti OR 'SARS-CoV-19':ab,ti OR 'SARS-CoV-2019':ab,ti OR 'SARS-CoV-2':ab,ti OR 'SARS2':ab,ti OR '2019-nCoV':ab,ti | 250,925 |
| 3 | 1 OR 2 | 270,074 |
| 4 | 'randomized controlled trial'/exp | 703,537 |
| 5 | 'randomized controlled trial (topic)'/exp | 222,499 |
| 6 | 'case control study'/exp | 202,934 |
| 7 | 'cohort analysis'/exp | 818,348 |
| 8 | 'cross-sectional study'/exp | 468,911 |
| 9 | 'randomized controlled trial':ab,ti OR 'controlled clinical trial':ab,ti OR 'randomized':ab,ti OR 'placebo':ab,ti OR 'randomly':ab,ti OR 'trial':ab,ti OR 'clinical research':ab,ti OR 'clinical observation':ab,ti OR 'control':ab,ti OR cohort*:ab,ti OR prospective:ab,ti OR longitudinal:ab,ti OR 'follow up':ab,ti OR 'case control':ab,ti OR 'case referent':ab,ti OR 'case stud*':ab,ti OR 'case series':ab,ti OR 'cross sectional' | 8,565,088 |
| 10 | 4-9/OR | 8,858,076 |
| 11 | 10 AND 3 [medline]/lim NOT ([embase classic]/lim AND [medline]/lim) AND [01-01-2019]/sd NOT [20-03-2022]/sd | 48,654 |

**Web of Science** 2019/01/01-2022/03/19

Search Strategy:

| **#** | **Searches** | **Results** |
| --- | --- | --- |
| 1 | TS=(coronavirus OR "corona virus" OR coronavirinae OR coronaviridae OR betacoronavirus OR covid19 OR "covid 19" OR nCoV OR "CoV 2" OR CoV2 OR sarscov2 OR 2019nCoV OR "novel CoV" OR "wuhan virus" OR "COVID-19" OR "COVID-2019" OR "SARS-CoV-19" OR "SARS-CoV-2019" OR "SARS-CoV-2" OR "SARS2" OR "2019-nCoV") | 282,624 |
| 2 | TS=("randomized controlled trial" OR "controlled clinical trial" OR "randomized" OR "placebo" OR "randomly" OR "trial" OR "clinical research" OR "clinical observation" OR "control" OR cohort* OR prospective OR longitudinal OR "follow up" OR "case control" OR "case referent" OR "case stud*" OR "case series" OR "cross sectional") | 9,033,609 |
| 3 | 1 AND 2 AND 2019/01/01-2022/03/19 | 65,572 |

**Cochrane Library** 2019/01/01-2022/03/19

Search Strategy:

| **#** | **Searches** | **Results** |
| --- | --- | --- |
| 1 | MeSH descriptor: [Coronavirus] this term only | 4 |
| 2 | MeSH descriptor: [Coronavirus Infections] this term only | 678 |
| 3 | MeSH descriptor: [Betacoronavirus] this term only | 127 |
| 4 | (coronavirus):ti,ab,kw OR ("corona virus"):ti,ab,kw OR (coronavirinae):ti,ab,kw OR (coronaviridae):ti,ab,kw OR (betacoronavirus):ti,ab,kw OR (covid19):ti,ab,kw OR ("covid 19"):ti,ab,kw OR (nCoV):ti,ab,kw OR ("CoV 2"):ti,ab,kw OR (CoV2):ti,ab,kw OR (sarscov2):ti,ab,kw OR (2019nCoV):ti,ab,kw OR ("novel CoV"):ti,ab,kw OR ("wuhan virus"):ti,ab,kw OR ("COVID-19"):ti,ab,kw OR ("COVID-2019"):ti,ab,kw OR ("SARS-CoV-19"):ti,ab,kw OR ("SARS-CoV-2019"):ti,ab,kw OR ("SARS-CoV-2"):ti,ab,kw OR ("SARS2"):ti,ab,kw OR ("2019-nCoV"):ti,ab,kw | 9,963 |
| 5 | 1-4/OR | 9,963 |
| 6 | MeSH descriptor: [Randomized Controlled Trial] explode all trees | 119 |
| 7 | MeSH descriptor: [Case-Control Studies] explode all trees | 14,490 |
| 8 | MeSH descriptor: [Cohort Studies] explode all trees | 158,377 |
| 9 | MeSH descriptor: [Controlled Before-After Studies] explode all trees | 86 |
| 10 | MeSH descriptor: [Cross-Sectional Studies] explode all trees | 5,023 |
| 11 | MeSH descriptor: [Historically Controlled Study] explode all trees | 22 |
| 12 | ("randomized controlled trial"):ti,ab,kw OR ("controlled clinical trial"):ti,ab,kw OR ("randomized"):ti,ab,kw OR ("placebo"):ti,ab,kw OR ("randomly"):ti,ab,kw OR ("trial"):ti,ab,kw OR ("clinical research"):ti,ab,kw OR ("clinical observation"):ti,ab,kw OR ("control"):ti,ab,kw OR (cohort*):ti,ab,kw OR (prospective):ti,ab,kw OR (longitudinal):ti,ab,kw OR ("follow up"):ti,ab,kw OR ("case control"):ti,ab,kw OR ("case referent"):ti,ab,kw OR ("case stud*"):ti,ab,kw OR ("case series"):ti,ab,kw OR ( "cross sectional"):ti,ab,kw | 1,464,394 |
| 13 | 6-12/OR | 1,465,230 |
| 14 | 5 AND 13 | 8,959 |
| 15 | 14 limited 2019/01/01-2022/03/19 | 8,901 |

**LOVE** 2019.01.01-2022.03.19

Search Strategy:

| **#** | **Searches** | **Results** |
| --- | --- | --- |
| 1 | "randomized controlled trial" OR "controlled clinical trial" OR "randomized" OR "placebo" OR "randomly" OR "trial" OR "clinical research" OR "clinical observation" OR "control" OR cohort* OR prospective OR longitudinal OR "follow up" OR “case control” OR “case referent” OR “case stud*” OR “case series” OR “cross sectional” | 3,492 |

**Table S2 Detailed guidance for assessment of risk of bias**

**Cochrane tool for assessing risk of bias in randomized clinical trials (RoB 2.0)**

| **Bias from the randomization process** | |
| --- | --- |
| Issues to consider:  Random sequence generation  Allocation concealment | |
| **Definitely low risk of bias** | Trials that assign participants to alternative interventions using a randomly generated sequence and maintain allocation concealment.  Examples of methods for developing a randomly generated allocation sequence include a random number generator, random number table, coin tossing, shuffling cards or envelopes, and throwing dice. If a trial is described as 'randomized' without any additional details related to how the allocation sequence was developed, we will assume that the allocation sequence was appropriately developed.  Examples of methods for maintaining allocation concealment include using central allocation via a computer or phone system, pharmacy-controlled allocation, opaque sealed envelopes, and sequentially numbered drug containers.  *Note that an explicit description of random sequence generation is not necessary for a rating of low risk of bias.* |
| **Probably low risk of bias** | Trials in which healthcare providers were blind to the intervention but which provide no information on allocation concealment and in which there are no major baseline imbalances.  *Note that an explicit description of random sequence generation is not necessary for a rating of probably low risk of bias.* |
| **Probably high risk of bias** | Trials in which healthcare providers were not blind to the intervention and which provide no information on allocation concealment.  Trials in which there are substantial baseline differences between trial arms that suggest a problem with the randomization process but there are no other limitations related to randomization. |
| **Definitely high risk of bias** | Trials in which allocation is by judgment of the clinician, by preference of the participant, by availability of the intervention, based on the results of a laboratory test, or other non-random rules (e.g., birthdate, etc.).  Trials in which investigators enrolling participants could possibly foresee the arm to which each subsequent patient would be randomized, such as allocation using an open allocation schedule (e.g. a list of random numbers), assignment envelopes used without appropriate safeguards (e.g. use of unsealed, non-opaque or not sequentially numbered envelopes), alternation between arms, case record number, or any other explicitly unconcealed procedure, rate as high risk. |
| **Bias due to deviations from the intended intervention** | |
| Issues to consider:  Blinding of healthcare providers/clinicians and participants  Imbalances in cointerventions or behaviors | |
| **Definitely low risk of bias** | Therapy trials in which healthcare providers are blind to the intervention administered and in which there are no significant differences in administered co-interventions.  Therapy trials that are described as double or triple blind.  Prophylaxis trials in which participants are blind to the intervention that they have been randomized.  Prophylaxis trials that are described as double or triple blind. |
| **Probably low risk of bias** |  |
| **Probably high risk of bias** | Therapy trials in which healthcare providers are not blind to the intervention administered.  Therapy trials in which healthcare providers are blind to the intervention administered but there are significant differences in administered co-interventions that suggests that blinding may have been compromised.  Therapy trials in which healthcare providers are described as being blind to the intervention but allocation concealment was inadequate.  Prophylaxis trials in which participants are not blind to the intervention that they have been randomized.  Prophylaxis trials in which participants are blind to the intervention to which they have been randomized but there are significant differences in social distancing and risk-taking behaviors that suggest that blinding may have been compromised.  Prophylaxis trials in which healthcare providers are not blind to the intervention and in which healthcare providers were very involved and counselled patients on social distancing, risk-taking behaviors, or testing for COVID-19. |
| **Definitely high risk of bias** | Therapy trials in which healthcare providers are not blind to the intervention and in which there are significant differences in administered co-interventions.  Prophylaxis trials in which participants are not blind to the intervention and in which there are significant differences in social distancing and risk-taking behaviors. |
| **Bias due to missing data** | |
| Issues to consider:  Missing outcome measures  Loss to follow-up | |
| **Definitely low risk of bias** | Trials in which missing outcome data (including outcome data that has been imputed) < 10%.  For in-patient trials, we will assume low risk of bias due to missing data unless otherwise specified. |
| **Probably low risk of bias** | Trials in which missing outcome data (including outcome data that has been imputed) is between 10% to 15% and missing outcome data is unlikely to be related to the true outcome and there is no imbalance in numbers of or reasons for missing data across intervention groups. |
| **Probably high risk of bias** | Trials in which missing outcome data (including outcome data that has been imputed) is between 10% to 15% and missing outcome data is likely to be related to the true outcome or there are imbalances in numbers of or reasons for missing data across intervention groups. |
| **Definitely high risk of bias** | Trials in which missing outcome data (including outcome data that has been imputed) > 15%. |
| **Bias due to measurement of the outcome** | |
| Issues to consider:  Blinding of outcome adjudicators  Objectivity of outcome  *Note that the judgments may differ across outcomes.* | |
| **Definitely low risk of bias** | Trials in which patients are blind to the intervention and in which outcomes are patient-reported.  Trials in which outcomes are measured by a third-party (investigator or clinician) and in which the third-party is blind to the intervention.  Trials in which the outcomes are objective (e.g., mortality, infection with COVID-19 confirmed by a positive RT-PCR swab, mechanical ventilation, admission to hospital, duration of hospital stay, ICU length of stay, ventilator free days, duration of mechanical ventilation, time to clinical improvement if clinical improvement is measured via objective criteria, viral clearance, time to viral clearance).  Trials that are described as double or triple blind. |
| **Probably low risk of bias** |  |
| **Probably high risk of bias** |  |
| **Definitely high risk of bias** | Trials in which patients are not blind and in which outcomes are patient-reported (e.g., time to symptom resolution).  Trials in which outcome adjudicators are not blind and the outcomes are not objective (e.g., adverse effects leading to discontinuation, transfusion-related acute lung injury, transfusion-associated circulatory overload, allergic reactions, infection with suspected/symptomatic COVID-19, venous thromboembolism, time to symptom resolution including fever, time to clinical improvement if the criteria for clinical improvement are not objective). |
| **Bias in selection of the reported results** | |
| Issues to consider:  Selective reporting of timepoints  Selective reporting of outcome measures  *Note that we are only interested in selective reporting for the outcomes for which we are extracting data.*  *Note that the judgments may differ across outcomes.* | |
| **Definitely low risk of bias** | Results for outcomes that were analyzed and reported according to a pre-specified statistical analysis plan or protocol (including the timepoint for the measurement of the outcome). |
| **Probably low risk of bias** | Results for outcomes that were analyzed and reported but that were not prespecified in a statistical analysis plan or protocol but the timepoint at which results are reported is consistent with the timepoint for other outcomes in the trial report or there is little reason to believe the outcome was selectively reported.  Please note that outcomes that were not prespecified in a protocol or statistical analysis plan and that are reported in the trial preprint or publication should be rated at probably low risk of bias unless there are other important reasons to suspect that results for those outcomes were selectively reported (e.g., results are presented at timepoints that don’t match the timepoints reported for other outcomes). |
| **Probably high risk of bias** | Results for outcomes that were analyzed and reported but that were not prespecified in a statistical analysis plan or protocol but the timepoint at which results are reported is not consistent with the timepoint for other outcomes in the trial report or there are other reasons to believe that the outcome is selectively reported. |
| **Definitely high risk of bias** | Results for outcomes that were analyzed and reported for which there are inconsistencies with the statistical analysis plan or protocol. These inconsistencies may include outcome measures of interest or the timepoints for the measurement of outcomes. |
| **Other sources** | |
| Issues to consider:  Other important sources of bias | |
| **Definitely low risk of bias** | Trials appear to be free of other important sources of bias.  For example:  The trial plan or protocol is prospectively registered on the clinical trial registration platform.  The trials are approved by the ethics committee.  The results are written up following the reporting guidelines. |
| **Definitely high risk of bias** | There is at least one important risk of bias.  For example:  No information on the prospective registration and the ethics committee approve.  Incomplete reporting of results or the important information is missing. |

**Table S3 Characteristics of included studies**

| **Study** | **Journal, JCR** | | **Language, registration No.** | | **N** | | **Timeline of patient recruitment** | | **Recruitment location** | | **Mean age (ys)** | **Men (%)** | **Comorbidities** | **Severity** |
| --- | --- | --- | --- | --- | --- | --- | --- | --- | --- | --- | --- | --- | --- | --- |
| RCT | |  | |  | |  | |  | |  | |  |  |  |
| Wen L  2020 | Chinese Critical Care Medicine, PKU | | Chinese, ChiCTR2000029381 | | 60 | | January to March 2020 | | Changsha, Hunan Province, China | | 47.97±5.29 | 53.3 | NR | 100% severe |
| Liu YJ  2021 | Laboratory Medicine and Clinic | | Chinese, NR | | 50 | | January to March 2020 | | Yichang, Hubei Province, China | | 48.25±1.49 | 54.00 | NR | 100% severe |
| Hu F  2020 | Pharmacological Research - Modern Chinese Medicine, Q1 | | English, ChiCTR2000030704 | | 40 | | From February 5, 2020 to March 5, 2020 | | Wuhan, Hubei Province, China | | 61.17±15.38 | 60.00 | Respiratory condition (2.50%), Cardiovascular disease or coronary heart disease (7.50%), Diabetes (22.50%), Hypertension (30.00%) | 100% severe |
| Luo ZJ  2021 | European Journal of Integrative Medicine, Q4 | | English, ChiCTR-2000030388 | | 57 | | From February 16  to March 25 in 2020 | | Jingzhou, Hubei Province, China | | 58.34±17.30 | NR | Cardiovascular disease or coronary heart disease (5.26%), Diabetes (14.04 %), Hypertension (52.63 %) | 100% severe |
| Liu ST  2021 | Integrative Medicine Research, Q3 | | English, ChiCTR2000029994 | | 128 | | From February 19 to March 31, 2020 | | Huangshi, Hubei Province, China | | 51.80±4.71 | 42.970 | Respiratory condition (0.78%), Cardiovascular disease or coronary heart disease (5.47%), Diabetes (10.16%), Hypertension (15.63%) | 100% severe |
| Zhou S  2021 | Phytomedicine, Q1 | | English, ChiCTR2000029777 | | 111 | | From January 14, 2020 to  March 28, 2020 | | 4 medical centers in Hubei Province,  China | | 64.67±12.14 | 63.96 | NR | 32.43% severe, 67.57% critical |
| Observational Studies | |  | |  | |  | |  | |  | |  |  |  |
| Zhang P  2021 | World Science and Technology-Modernization of Traditional Chinese Medicine, PKU | | Chinese, NR | | 24 | | From March 5, 2020 to April 5, 2020 | | Wuhan, Hubei Province, China | | 62.84 ± 14.69 | 54.17 | NR | 75.00% severe, 25.00% critical |
| Qin YL  2020 | China Pharmaceuticals | | Chinese, NR | | 47 | | January to March 2020 | | Chongqing, China | | 58.17±2.97 | 53.19 | Respiratory condition (2.13%), Cardiovascular disease or coronary heart disease (6.38%), Diabetes (29.79%), Hypertension (17.02) | 100% severe/critical |
| Wang Y  2021 | Journal of Ethnopharmacology, Q1 | | English, NR | | 55 | | From January 17th, 2020 to February 25th, 2020; | | Wuhan, Hubei Province, China | | 55.83±17.89 | 52.70 | Respiratory condition (3.60%), Cardiovascular disease or coronary heart disease (23.60%) | 100% severe |
| Zhang LH  2020 | Phytomedicine, Q1 | | English, NR | | 8,939 | | January to May 2020 | | 15 hospitals in Hubei Province, China | | 55.90 ± 15.60 | 46.60 | Respiratory condition (1.76%), Cardiovascular disease or coronary heart disease (7.67%), Diabetes (15.20%), Hypertension (33.70%) | 4.4% critical |
| Chen L  2021 | Chinese Critical Care Medicine, PKU | | Chinese, NR | | 65 | | January to March 2020 | | Chongqing, China | | 61.89±13.51 | 50.77 | NR | 100% severe/critical |
| Liu XS  2021 | Chinese Critical Care Medicine, PKU | | Chinese, ChiCTR 2000029381 | | 144 | | January to March 2020 | | 28 hospitals in 15 provinces and cities | | 64.60±9.50 | 72.90 | Cardiovascular disease or coronary heart disease (15.30%), Diabetes (24.30%), Hypertension 48.60%) | 100% severe/critical |
| Feng J  2021 | Journal of Microbiology and Biotechnology, Q4 | | English, NR | | 118 | | From January 28 to March 28, 2020 | | Wuhan, Hubei Province, China | | 66.67±13.66 | 67.10 | Cardiovascular disease or coronary heart disease (17.70%), Diabetes (21.10%), Hypertension 44.00%) | 100% critical |
| Hu HB  2021 | Frontiers in Pharmacology | | English, NR | | 81 | | From February 9th to  March 31st, 2020, | | Wuhan, Hubei Province, China | | 64.66±8.43 | 43.75 | Cardiovascular disease or coronary heart disease (20.31 %), Diabetes (21.88 %), Hypertension (29.69 %) | 100% severe |
| Huang DH  2021 | Shanxi Journal of Traditional Chinese Medicine, Q1 | | Chinese, NR | | 50 | | From February 1, 2020 to May 31, 2020 | | Wuhan, Hubei Province, China | | 58.84±5.12 | 60.00 | NR | 100% severe |
| Xiong YB  2022 | Phytomedicine, Q1 | | English, NR | | 111 | | From January 24, 2020, to March 31, 2020 | | Wuhan, Hubei Province, China | | 61.81±12.71 | 67.60 | Respiratory condition (7.20%), Cardiovascular disease or coronary heart disease (44.10%) | 100% severe |
| Chen GH  2020 | Frontiers of Medicine, Q2 | | English, ChiCTR2000030719 | | 662 | | Before March 20, 2020. | | Wuhan, Hubei Province, China | | 62±15.64 | 45.50 | NR | 75.60% severe, 24.40% critical |
| Wang YY  2021 | Phytomedicine, Q1 | | English, NR | | 130 | | From January 15, 2020 to March 30, 2020 | | Wuhan, Hubei Province, China | | 64.17±9.80 | 45.00 | Respiratory condition (37.00%), Cardiovascular disease or coronary heart disease (19.00%), Diabetes (16.00%), Hypertension 41.00%) | 100% severe |
| Shu ZX  2021 | The American Journal of Chinese Medicine, Q1 | | English, NR | | 1788 | | From 15 January to 13 March 2020 | | 5 hospitals in Wuhan, China | | 70.92±14.26 | 54.84 | Cardiovascular disease or coronary heart disease (12.90%), Diabetes (12.90%), Hypertension 35.48%) | 17.73% severe/critical |
| Sun QG  2021 | The American Journal of Chinese Medicine, Q1 | | English, ChiCTR2000031187 | | 282 | | From January 27, 2020, to March 20, 2020 | | Wuhan, Hubei Province, China | | 68.67±13.45 | 55.90 | Cardiovascular disease or coronary heart disease (16.5%), Diabetes (19.10%), Hypertension 48.40%) | 61.20% severe, 38.8% critical |
| Zhao J  2020 | Biomedicine & Pharmacotherapy, Q1 | | English, NR | | 39 | | January 20, 2020 to February 24, 2020 | | Hefei, Anhui Province, China | | NR | 56.41 | NR | 100% severe |

**Table S4 Studies reporting treatment of COVID-19 patients**

| Study | TCMs（dose and duration） | TCM components | Conventional treatment |
| --- | --- | --- | --- |
| RCT | | | |
| Wen L | XBJ (50mL/100mL, 2 times a day for 7 days) | XBJ: five herbs extraction, which are Carthamus tinctorius Linn (Honghua), Paeoniae Radix rubra (Chishao), Chuanxiong Rhizoma (Chuanxiong), Angelicae Sinensis Radix (Danggui), and Salviae Miltiorrhizae (Danshen) | NHC-China Guidelines: general treatment |
| Liu YJ | HSBD (1-2 doses/day, 100-200mL/time, 2-4 times/day) | HSBD: Ephedrae Herba (Shengmahuang), 6 g; Armeniacae Semen (Xingren), 9 g; Gypsum Fibrosum (Shengshigao), 15 g; Glycyrrhizae Radix (Gancao), 3 g; Pogostemonis Herba (Huoxiang), 10 g; Magnoliae Officmalis Cortex (Houpo), 10 g; Atractlodis Rhizoma (Cangzhu), 15 g; Tsaoko Fructus (Caoguo), 10 g; Pinellinae Rhizoma Praeparatum (Fabanxia), 9 g; Poria (Fuling), 15 g; Rhei Radix et Rhizoma (Shengdahuang), 5 g; Astmgali Radix (Shenghuangqi), 10 g; Descurainiae Semen (Tinglizi), 10 g; Paeoniae Radix rubra (Chishao), 10 g. | Tocilizumab [the first dose of 4-8 mg/kg, the recommended dose is 400 mg, diluted with normal saline to 100 mL for intravenous infusion, and the infusion time is more than 1 h; if the efficacy of the first treatment is not good, it can be additionally applied after 12 h (dose The same as before), the cumulative number of daily doses is at most 2 times, the maximum single dose does not exceed 800 mg, and 7-10 days are regarded as a treatment cycle]. At the same time, oxygen therapy, mechanical ventilation, and plasma therapy for recovered patients were given according to the patient's condition. |
| Hu F | CS (20 mL/day, containing about 60μg dry extract of Chansu) | SC: bufotalin bufalin, cinobufogenin, resibufogenin | General treatment includes, as necessary, empirical antiviral therapy with peramivir, arbidol and interferon α, nutritional support, supplemental oxygen and standard glucocorticoid therapy, etc |
| Luo ZJ | XBJ (50ml diluted with 100 ml normal saline to 150 ml, every 12h for 14 days) | XBJ: five herbs extraction, which are Carthamus tinctorius Linn (Honghua), Paeoniae Radix rubra (Chishao), Chuanxiong Rhizoma (Chuanxiong), Angelicae Sinensis Radix (Danggui), and Salviae Miltiorrhizae (Danshen) | Nutritional support, oxygen therapy, antiviral therapy with interferon-a inhalation, antibiotic agents, non-invasive and invasive ventilation if necessary |
| Liu ST | QARP was performed twice daily at 10 am and 4 pm. The participants were required to pronounce "Xu, He, Hu, Si, Chui, Xi” in a relaxed condition for 12 cycles. Guideline workbooks and videos of qigong exercise were used to facilitate subsequent practice. Each treatment lasted 20 min for a total of 40 min every day. The acupressure treatment was performed by a physical therapist every day after reaching a consensus on the participants’ acupuncture points, pressure levels, and duration. The therapist pressed the acupoints with moderate force when the participants were in the sitting or supine position, with the pressure direction perpendicular to the skin surface for 3–7 s. The acupoints were Feishu (BL13), Danzhong (RN17), and Zhongfu (LU1), which are related to the lung viscera. The therapy was continued during the patient’s stay until the day of discharge | | Drugs include antiviral therapy α-interferon (5 million U, twice a day, aerosol inhalation) + ribavirin (500 mg, 2-3 times a day, intravenous infusion), glucocorticoid treatment (methylprednisolone 0.5∼1 mg/kg/day), anticoagulant therapy (the recommended dose of subcutaneous injection of low molecular weight heparin was 100 UK/q12h for 3-5 days) and tozumab (4∼8 mg/kg for the first dose, 400 mg for the recommended dose, cumulative number of doses up to 2 times, and the maximum dose for a single dose not exceeding 800 mg). Doctors should prescribe antiviral drugs combined with antibiotics if necessary. Timely effective oxygen therapy measures should be taken |
| Zhou S | SH (2sachets/day,14-day) | SH:50 g of Panax ginseng C. A. Mey (Renshen) root, 40 g of Rheum palmatum L. stem (Dahuang), 30g of Sargentodoxa cuneata stem (Hongteng), 30 g of Taraxacum mongolicum whole plant (Pugongying), 50 g of Aconiti Lateralis Radix Praeparata stem (Fuzi) and 6g of Whitmania pigra Whitman (Shuizhi) whole organism. | NHC-China Guideline (Interim Version 7) |
| Observational Studies | | | |
| Zhang P | QFPD (Decoction of 400 mL for 1 dose per day, 200Ml/time, bid for 7 days) | QFPD: Herba Ephedrae (Mahuang)9g, Radixet Rhizoma Glycyrrhizae Praeparata cum Melle (Zhigancao) 6g, Semen Armeniacae Amarum (xinren) 9g, Gypsum Fibrosum (Shengshigao) 15–30 g, , Ramulus Cinnamomi 9g (Guizhi), Rhizoma Alismatis (Zexie) 9 g, Polyporus (Zhuling) 9g, Rhizoma Atractylodis Macrocephalae (Baizhu) 9g, Poria(Fuling) 15g, Radix Bupleuri (Chaihu) 16g, Radix Scutellariae (Huangqin) 6g, Rhizoma Pinelliae Praeparatum (Jiangbanxia) 9g, Rhizoma Zingiberis Recens (Shengjiang) 9g, Radix et Rhizoma Asteris (Ziwan) 9 g, Flos Farfarae (Kuandonghua) 9g, Rhizoma Belamcandae (Shegan) 9 g, Radix et Rhizoma Asari (Xixin) 6g, Rhizoma Dioscoreae (Shanyao) 12 g, Fructus Aurantii Immaturus (Zhishi) 6g, Pericarpium Citri Reticulatae (Chenpi) 6g, and Herba Agastachis (Huoxiang)9 g. | Arbidol hydrochloride tablets (0.2 g, bid, for 7 days);  Anti-infection: Moxifloxacin hydrochloride (0.4 g, qd, for 7 days). Adjust antibiotics and their course of treatment according to changes in the condition.  Vitamin C (0.2 g, tid, for 14 days)  Symptomatic support: oxygen therapy, phlegm reduction, nutrition and respiratory support, etc.  α-Interferon: 5 million units of IFNα, add 2 mL of sterile water for injection, inhalation, bid |
| Qin YL | RDN (20 mL intravenous drip, once a day for 5-7 days) | Flos Lonicerae Japonicae (Jinyinhua), Gardeniae Fructus (Zhizi)，Artemisia annuae herba (Qinghao) | Methylprednisolone powder injection (1 ~ 2 mg/kg·d intravenous infusion, once a day for 3 ~ 5 days) |
| Wang Y | HSBD (137 g po, bid) combined with the XYP(100 mg iv, bid), XBJ (100 ml iv, bid) and SM (60 ml iv, qd) according to the syndrome of epidemic toxin blocking the lung in the theory of TCM. | HSBD: Ephedrae Herba (Shengmahuang), 6 g; Armeniacae Semen (Xingren), 9 g; Gypsum Fibrosum (Shengshigao), 15 g; Glycyrrhizae Radix (Gancao), 3 g; Pogostemonis Herba (Huoxiang), 10 g; Magnoliae Officmalis Cortex (Houpo), 10 g; Atractlodis Rhizoma (Cangzhu), 15 g; Tsaoko Fructus (Caoguo), 10 g; Pinellinae Rhizoma Praeparatum (Fabanxia), 9 g; Poria (Fuling), 15 g; Rhei Radix et Rhizoma (Shengdahuang), 5 g; Astmgali Radix (Shenghuangqi), 10 g; Descurainiae Semen (Tinglizi), 10 g; Paeoniae Radix rubra (Chishao), 10 g.  XYP: (SFDA approval number Z20026249, 50mg/ piece) was made by sulfonation process of andrographis B extracted from Andrographis Herba (Chuanxinlian), which was provided by Jiangxi Qingfeng Pharmaceutical Co., Ltd. (Ganzhou, China).  XBJ: (SFDA approval number Z20040033 for 10ml/ piece) is a combination of five herbs extraction, which are Carthamus tinctorius Linn (Honghua), Paeoniae Radix rubra (Chishao), Chuanxiong Rhizoma (Chuanxiong), Angelicae Sinensis Radix (Danggui), and Salviae Miltiorrhizae (Danshen), which was provided by Tianjin Chase Sun Pharmaceutical Co. Ltd. (Tianjin, China).  SM: (SFDA approval number Z33020020 for 20ml/ piece) was made from Red Ginseng (Hongshen) and Radix Ophiopogonis (Maidong), which was provided by Chiatai Qingchunbao Pharmaceutical Co., Ltd. (Hangzhou, China). | Antiviral therapy (including abider capsule 0.2 g po, tid; Lopinavir–Ritonavir tablets, 500 mg po, bid), antibiotics (such as cefoperazone, 2 g iv, bid; moxifloxacin hydrochloride tablets, 0.4 g po, qd) or corticosteroid therapy (such as methylprednisolone succinate sodium 40 mg iv, qd; prednisone, 30 mg po, qd). Supportive therapy in both groups included oxygen inhalation, symptomatic treatment, and/or human intravenous immunoglobulin, and/or serum albumin, and treatment for underlying diseases. |
| Zhang LH | QPTD (no less than three days) | Herba Ephedrae (Mahuang)9g, Radixet Rhizoma Glycyrrhizae Praeparata cum Melle (Zhigancao) 6g, Semen Armeniacae Amarum (xinren) 9g, Gypsum Fibrosum (Shengshigao) 15–30 g, , Ramulus Cinnamomi 9g (Guizhi), Rhizoma Alismatis (Zexie) 9 g, Polyporus (Zhuling) 9g, Rhizoma Atractylodis Macrocephalae (Baizhu) 9g, Poria(Fuling) 15g, Radix Bupleuri (Chaihu) 16g, Radix Scutellariae (Huangqin) 6g, Rhizoma Pinelliae Praeparatum (Jiangbanxia) 9g, Rhizoma Zingiberis Recens (Shengjiang) 9g, Radix et Rhizoma Asteris (Ziwan) 9 g, Flos Farfarae (Kuandonghua) 9g, Rhizoma Belamcandae (Shegan) 9 g, Radix et Rhizoma Asari (Xixin) 6g, Rhizoma Dioscoreae (Shanyao) 12 g, Fructus Aurantii Immaturus (Zhishi) 6g, Pericarpium Citri Reticulatae (Chenpi) 6g, and Herba Agastachis (Huoxiang)9 g. | those not receiving QPT |
| Chen L | XBJ was applied to patients with elevated inflammatory cytokines, and human immunoglobulin was applied to patients whose lymphocytes and their subpopulation monitoring results indicated that the immune system was low. | XBJ: five herbs extraction, which are Carthamus tinctorius Linn (Honghua), Paeoniae Radix rubra (Chishao), Chuanxiong Rhizoma (Chuanxiong), Angelicae Sinensis Radix (Danggui), and Salviae Miltiorrhizae (Danshen) | Conventional antiviral (interferon aerosol inhalation, lopinavir/ritonavir tablets orally or nasal feeding), anti-infection and symptomatic supportive treatment |
| Liu XS | XBJ (100 mL/time, 2 times/day, the course of treatment should not be less than 1 d) | XBJ: five herbs extraction, which are Carthamus tinctorius Linn (Honghua), Paeoniae Radix rubra (Chishao), Chuanxiong Rhizoma (Chuanxiong), Angelicae Sinensis Radix (Danggui), and Salviae Miltiorrhizae (Danshen) | NHC-China Guideline: Including antiviral, respiratory support, circulatory support, symptomatic treatment, and other medical care measures deemed appropriate by the competent physician. |
| Feng J | SH (Two sachets/day) | SH: 50 g of Panax ginseng C. A. Mey, 40 g of Rheum palmatum L. stem, 30 g of Sargentodoxa cuneata stem, 30 g of Taraxacum mongolicum, 50 g of Aconiti Lateralis Radix Praeparata and 6 g of Whitmania pigra Whitman | NHC-China Guideline (7^th^ edition)The standard care included timely provision of effective oxygen therapy, circulatory support, renal failure and renal replacement therapy, blood purification treatment, immunotherapy and other therapeutic measures such as short-term use of glucocorticoids. |
| Hu HB | HJSS (per os twice daily) | HJSS:Bupleurum chinense DC.18g;Scutellaria baicalensis Georgi 9g; Pinellia ternata (Thunb.) Makino 9g; Glycyrrhiza uralensis Fisch.ex DC. 6g; Codonopsis pilosula (Franch.) Nannf. 9g; Poria cocos (Schw.)Wolf 18g;Alisma plantago-aquatica subsp. orientale (Sam.) Sam.12g;Atractylodes macrocephala Koidz. 12g;Neolitsea cassia (L.) Kosterm. 6g;Coix lacryma-jobi var. ma-yuen (Rom.Caill.) Stapf 18g;Pyrrosia lingua (Thunb.) Farw. 12g;Plantago asiatica L. 12g;Benincasa hispida (Thunb.) Cogn. 15g. | oxygen inhalation therapy (high-flow nasal cannula oxygen therapy and mechanical ventilation if necessary), parenteral nutrition, antiviral therapy (arbidol, 200 mg, per os three times daily) and other treatments based on the NHC-China Guideline (4th–6th editions). |
| Huang DH | GZ 3 (2 doses/day, once in the morning and once in the evening) | Corni Fructus (Shanzhuyu)45 g，Os Draconis (Shenglonggu) 30 g、Ostreae Concha(Muli) 30 g，Cooked Aconite (Shufuzi) 20 g、Ginseng Radix ET Rhizoma (Renshen) 20 g、Magnetitum (powder) (Cisifen)20 g， Rhizoma Zingiberis (Ganjiang)、Radix et Rhizoma Glycyrrhizae Preparata (Zhigancao)、Atractylodis Macrocephalae Rhizoma (Fired) (Chaobaishu) 15g、Poria (Fuling) 15 g，Acori Tatarinowii Rhizoma (Shichangpu) 12 g, Schisandrea Chinensis Frutus (Wuweizi) 9 g | Both groups of patients were given routine bed rest, non-invasive mechanical ventilation, nutritional support, immune support and other treatments, and invasive ventilation when necessary. The western medicine group received antiviral treatment: Arbidol hydrochloride tablets, 200 mg each time, 3 times a day orally, the course of treatment did not exceed 10 days; antibacterial treatment: Moxifloxacin hydrochloride tablets, 400 mg each time, once a day Oral administration, the course of treatment should not exceed 7 days; adjuvant and supportive treatment: amino acid injection, 250 mL intravenously per day for 8 to 10 consecutive days, and human immune globulin if necessary, 400 mg/kg intravenously per day for 3 to 3 consecutive days 5 days. |
| Xiong YB | HSBD (Two or four formula a day, orally or by nasal feeding, at least 14 days for one course) | HSBD: Raw ephera, Agastache, Raw gymsum, Almond, Rhizoma Pinellinae Praeparata, Magnolia Officinalis, Rhizoma atractylodis, Amomum tsao-ko, Poria cocos, Radix Astragali, Radix Paeoniae Rubra, lepidium seed, rhubarb, and Liquorice | The use of antibiotics, antiviral drugs, hormones, other proprietary Chinese medicine preparations were all acceptable |
| Chen GH | MHLJ (orally 200 mL/time, twice a day in hospital days) | MHLJ: Fuling (Poriacocos (Schw.) Wolf.), Huangqi (Astragalus membranaceus(Fisch.) Bge. var. mongholicus (Bge.) Hsiao.), Huoxiang(Pogostemon cablin (Blanco) Benth.), Kuxingren (Prunusarmeniaca L. var. ansu Maxim.), Baizhu (Atractylodesmacrocephala Koidz.), Banxia (Pinellia ternata (Thunb.)Breit.), Gancao (Glycyrrhiza uralensis Fisch.), Houpo(Magnolia officinalis Rehd. et Wils.), Mahuang (Ephedrasinica Stapf), Guizhi (Cinnamomum cassia Presl), Huangqin (Scutellaria baicalensis Georgi.), Sharen (Amomumvillosum Lour.), Jiegeng (Platycodon grandiflorum (Jacq.)A.DC.), Peilan (Eupatorium fortunei Turcz.), andDangsheng (Codonopsis pilosula (Franch.) Nannf.) purchased from Hubei Tianji TCM Decoction Pieces Co., Ltd. | Patients were treated with oxygen, antivirals (such as interferon or ribavirin), antibiotics (such as moxifloxacin, cefoperazone sodium, and sulbactam sodium), and Chinese medicine based on the NHC-China (Trial Version). Critical patients were treated with noninvasive mechanical ventilation, invasive mechanical ventilation, and extracorporeal membrane oxygenation to support life |
| Wang YY | CHJD and FZJF (Oral or nasal feeding of 1–2 doses mixed with 100–200 mL water for 2 times a day) | Chai-hu-jie-du granule composition: Chaihu (Radix Bupleuri), 30 g; Huangqin (Radix Scutellaria), 15 g; Fabanxia (Rhizoma Pinelliae Praeparatum), 15 g; Shengjiang (Rhizoma Zingiberis Recens), 15 g; Dazao (Ziziphus jujuba Mill), 5 g; Zhishi (Fructus Aurantii Immaturus), 20 g; Dahuang (Radix et Rhizoma Rhei), 10 g; Taoren (Semen Persicae), and 10 g; Baishao (Radix Paeoniae Alba), 15 g.Administration and dosage: Oral or nasal feeding of 1–2 doses mixed with 100–200 mL waterfor 2 times a day.  Fu-zheng-jiu-fei granule composition: Zhifuzi (Radix Aconiti Lateralis Preparata), 10 g; Ganjiang (Rhizoma Zingiberis), 15 g; Zhigancao (Radix et Rhizoma Glycyrrhizae Preparata), 20 g; Jinyinhua (Flos Lonicera), 10 g; Zaojiaoci (Spina Gleditsiae), 10 g; Wuzhaolong (Radix Ficus Hirta), 20 g; Guanghuoxiang (Herba Pogostemonis), 10 g; Chenpi (Pericarpium Citri Reticulatae), 5 g. | According to the guidelines of the China’s guidelines, usual care comprised general treatment, supplemental oxygen, mechanical ventilation, antibiotic agents, and antiviral therapy |
| Shu ZX | Severe: 1.Cang-zhu 15g, chen-pi 10g, hou-pu 10g, huo-xiang 10g, cao-guo 6g, ma-huang 6g, qiang-huo 10g, sheng-jiang 10g, bin-lang 10g  2.Ma-huang 6g, xing-ren 9g, shi-gao 15g, gan-cao 3g, huo-xiang 10g, hou-pu 10g, cang-zhu 15g, cao-guo 10g, ban-xia 9g, fu-ling 15g, da-huang 5g, huang-qi 10g, ting-li-zi 10g, chi-shao 10g  3.Shi-gao 30–60g, zhi-mu 30g, di-huang 30–60g, shui-niu-jiao 30g, chi-shao 10g, xuan-shen 30g, lian-qiao 15g, mu-dan-pi 15g, huang-lian 6g, zhu-ye 12g, ting-li-zi 10g, gan-cao 6g  Critical:Ren-shen 15g, fu-zi 10g, shan-zhu-yu 15g with An-gong-niu-huang pill or Su-he-xiang pill | | Standard care |
| Sun QG | TCMD: QFPD, XSLJ, MXSG, Tongshi Wuhan Anti-epidemic Recipe, Bamboo Leaf Gypsum Decoction, Ganlu Sangui Decoction, Shashen Ophiopogon Decoction, Maxing Shigan Decoc tion, Rhubarb Huangqin Huanglian Decoction, Huopu Xialing Decoction, and Chaihuda Original DecoctionTCMDs were chosen on the basis of the patient’s specific condition. Most patients used 1–5 TCMDs during hospitalization. The decoction (400 ml, twice a day) was generally administered orally; however, it was administered nasally if patients were on invasive mechanical ventilation. | The main components of TCMD were determined on the basis of Maxingshigan Decoction (MXSGD): Timotherm, Gypsum, Ephedra, Bitter almond, Cassia, Astragalus, Pinellia, Ginger, Artemisia annua, Penang, and Licorice. The components were processed into decoctions by Wuhan Tianji Pharmaceutical Co., Ltd. The decoction (400 ml, twice a day) was generally administered orally; however, it was administered nasally if patients were on invasive mechanical ventilation. | According to the 7th edition of the guidelines, severely and critically ill patients must be treated symptomatically (general treatment, antiviral treatment, and antibacterial treatment), which involves active prevention and treatment of complications, treatment of basic diseases, and prevention of secondary infections in a timely manner; organ function support, respiratory support (including oxygen therapy, high-flow nasal catheter oxygen therapy or non-invasive mechanical ventilation, invasive mechanical ventilation, rescue therapy), and circulatory support; and renal failure and renal replacement therapy, plasma therapy for recovered patients, blood purification treatment, immunotherapy, and other treatment measures (including the use of glucocorticoids, methylprednisolone, intravenous injection of Xuebijing) |
| Zhao J | YDZF: received the 5th edition recommendation’s CM rescription extraorally for two weeks. | Recommendation of CM prescription: Kuxingren (Bitter Apricot Seed), 10g; Shengshigao (Calcium sulfate dihydrate), 30g; Gualou (Trichosanthes kirilowii Maxim), 30g; Shengdahuang (Radix et Rhizoma Rhei), 6g; Shengmahuang (Ephedra saxatilis Royle ex Florio), 6g; Ting li zi (Lepidium apetalum Willd), 10g; Taoren (Semen Persicae), 10g; Caoguo (Amomum tsaoko), 6g; Binglang (Arecae Semen), 10g; Cangzhu Atractylodis Rhizoma), 10g. | The general strategies were given to both groups according to the National recommendations for diagnosis and treatment of pneumonia caused by SARS-COV-2 (the 5th edition), including bed rest and supportive treatments; ensuring sufficient calories and water intake; maintaining water electrolyte balance and homeostasis, and strengthening psychotherapy for elder children when necessary. |

**Table S5 Risk of bias of included RCTs**

| **Study** | **Bias from the randomization process generated** | **Bias due to deviations from the intended intervention** | **Bias due to missing data** | **Bias due to measurement of the outcome** | **Bias in selection of the reported results** | **Other bias** |
| --- | --- | --- | --- | --- | --- | --- |
| Wen L | Probably high | High | Low | Low | High | Low |
| Liu YJ | High | High | Low | Probably low | Probably low | High |
| Hu F | Probably high | High | High | Low | Low | Low |
| Luo ZJ | Low | Low | Low | Low | Low | Low |
| Zhou S | Low | Probably low | Low | Probably low | Probably low | Low |
| Liu ST | Low | Probably high | Low | Probably low | Probably low | Low |

**Table S6 Risk of bias of included observational studies**

| **Study** | **Bias due to confunding** | **Bias in selection of participants into the study** | **Bias from the exposure** | **Bias due to missing data** | **Bias due to measurement of the outcome** | **Bias in selection of the reported results** |
| --- | --- | --- | --- | --- | --- | --- |
| Zhang P | High | Low | Low | Low | Low | Low |
| Qin YL | High | Low | Low | Low | Low | Low |
| Wang Y | High | Low | Low | Low | Low | Low |
| Zhang LH | Low | Low | Low | Low | Low | Low |
| Chen L | Probably High | Low | Low | Low | Low | Low |
| Liu XS | Low | Low | Low | Low | Low | Low |
| Feng J | High | Low | Low | Low | Low | Low |
| Hu HB | Low | Low | Low | Low | Low | Low |
| Huang DH | High | Low | Low | Low | Probably Low | Low |
| Xiong YB | High | Low | Low | Low | Low | Low |
| Chen GH | Low | Low | Low | Low | Low | Low |
| Wang YY | Low | Low | Low | Low | Probably Low | Low |
| Shu ZX | Low | Low | Probably Low | Low | Low | Low |
| Sun QG | Low | Low | Low | Low | Low | Low |
| Zhao J | High | Low | Low | Low | Low | Low |

**Table S7 Summary of findings on mortality**

| **Study** | **Type** | **TCM** | **Design** | **N** | **Mean age (ys)** | **Men (%)** | **Comorbidities** | **Treat vs. control** | **RR (95%CI)** |
| --- | --- | --- | --- | --- | --- | --- | --- | --- | --- |
| Luo ZJ 2021 | 100% severe | XBJ | RCT | 57 | 58.34±17.30 | NR | Cardiovascular disease or coronary heart disease (5.26%), Diabetes (14.04 %), Hypertension (52.63 %) | 3.45% vs. 25.00% | RR=0.14 [0.02, 1.05] |
| Zhou S  2021 | 32.43% severe, 67.57% critical | SH | RCT | 111 | 64.67±12.14 | 63.96 | NR | **Total: 38.6% vs. 75.9%**  **Severe: 5.3% vs. 58.8%**  **Critical: 55.3% vs. 83.8%** | **Total: RR=0.51 [0.35, 0.73]**  **Severe: RR=0.09 [0.01, 0.63]**  **Critical: RR=0.66 [0.48, 0.91]** |
| Zhang P 2021 | 75.00% severe, 25.00% critical | QFPD | Observational study | 24 | 62.84 ± 14.69 | 54.17 | NR | 0.00% vs. 16.67% | RR=0.20 [0.01, 3.77] |
| Zhang LH  2020 | 4.4% critical | QFPD | Observational study | 8,936 | 55.90 ± 15.60 | 46.60 | Respiratory condition (1.76%), Cardiovascular disease or coronary heart disease (7.67%), Diabetes (15.20%), Hypertension (33.70%) | **1.2% vs. 4.8%^a^** | **aHR=0.50 [0.37, 0.66]** |
| Liu XS  2021 | 100% severe/critical | XBJ | Observational study | 144 | 64.60±9.50 | 72.90 | Cardiovascular disease or coronary heart disease (15.30%), Diabetes (24.30%), Hypertension 48.60%) | 8.33% vs. 18.06% | RR=0.46 [0.19, 1.15] |
| Chen GH  2020 | 75.60% severe, 24.40% critical | MHLJ | Observational study | 662 | 62.00 | 45.50 | NR | **8.30% vs. 23.10% ^a^** | **RR=0.35 [0.19, 0.63]** |
| Wang YY 2021 | 100% severe | CHJD+FZJF | Observational study | 130 | 64.17 | 45.00 | Respiratory condition (37.00%), Cardiovascular disease or coronary heart disease (19.00%), Diabetes (16.00%), Hypertension 41.00%) | **5.00% vs. 21.00%** | **RR=0.22 [0.05, 0.97]** |
| Feng J 2021 | 100% critical | SH | Observational study | 118 | 66.67±13.66 | 67.10 | Cardiovascular disease or coronary heart disease (17.70%), Diabetes (21.10%), Hypertension 44.00%) | **45.40% vs. 80.00%** | **RR=0.57 [0.39, 0.84]** |
| Shu ZX  2021 | 17.73% severe/critical | semi-individualized | Observational study | 1788 | 70.92±14.26 | 54.84 | Cardiovascular disease or coronary heart disease (12.90%), Diabetes (12.90%), Hypertension 35.48%) | **3.8% vs. 17.0%** | **All: aHR = 0.42 [0.23, 0.77] ^a^**  **Severe: aHR = 0.34 [0.15, 0.76] ^a^** |
| Sun QG  2021 | 61.20% severe, 38.8% critical | TCM | Observational study | 282 | 68.67±13.45 | 55.90 | Cardiovascular disease or coronary heart disease (16.5%), Diabetes (19.10%), Hypertension 48.40%) | **11.80% vs. 40.60%** | **OR: 0.12 [0.04, 0.38]^a^** |
| Chen L  2021 | 100% severe/critical | XBJ | Observational study | 65 | 61.89 | 50.77 | NR | Accumulate survival rate: 92.86% vs. 86.96% | 1.07 [0.89, 1.28] |

a: Propensity score-matched

**Table S8 Summary of results of rate/time of mechanical ventilation**

| **Study** | **Type** | **TCM** | **Design** | **N** | **Mean age (ys)** | **Men (%)** | **Comorbidities** | **Treat vs. control** | **RR/MD (95%CI)** |
| --- | --- | --- | --- | --- | --- | --- | --- | --- | --- |
| Rate of mechanical ventilation | | | | | | | | | |
| Luo ZJ 2021 | 100% severe | XBJ | RCT | 57 | 58.34±17.30 | NR | Cardiovascular disease or coronary heart disease (5.26%), Diabetes (14.04 %), Hypertension (52.63 %) | **10.34% vs. 46.43%** | **RR= 0.22 [0.07, 0.70]** |
| Zhou S 2021 | 32.43% severe, 67.57% critical | SH | RCT | 111 | 64.67±12.14 | 63.96 | NR | **0% vs. 58.8%** | **RR=0.04 [0.00, 0.68]** |
| Qin YL 2020 | 100% severe/critical | RDN | Observational study | 47 | 58.17± 2.97 | 53.19 | Respiratory condition (2.13%), Cardiovascular disease or coronary heart disease (6.38%), Diabetes (29.79%), Hypertension (17.02) | 9.52% vs. 23.08% | RR=0.41 [0.09, 1.84] |
| Time of mechanical ventilation (days) | | | | | | | | | |
| Hu F 2020 | 100% severe | CS | RCT | 40 | 61.17±15.38 | 60.00 | Respiratory condition (2.50%), Cardiovascular disease or coronary heart disease (7.50%), Diabetes (22.50%), Hypertension (30.00%) | **5.00 vs. 6.33 days** | **MD= -1.33 [-2.61, -0.05]** |

a: Propensity score-matched

**Table S9 Summary of results of rate/time of the nucleic acid conversion**

| **Study** | **Type** | **TCM** | **Design** | **N** | **Mean age (ys)** | **Men (%)** | **Comorbidities** | **Treat vs. control** | **RR/MD (95%CI)** |
| --- | --- | --- | --- | --- | --- | --- | --- | --- | --- |
| Rate of the nucleic acid conversion | | | | | | | | | |
| Wen L 2020 | 100% severe | XBJ | RCT | 60 | 47.97±5.29 | 53.3 | NR | 42.50% vs. 45.00% | RR= 0.94 [0.52, 1.73] |
| Wang Y 2021 | 100% severe | HSBD+XYP+XBJ+SM | Observational study | 55 | 55.83±17.89 | 52.70 | Respiratory condition (3.60%), Cardiovascular disease or coronary heart disease (23.60%) | **30% vs. 6%** | **RR= 4.87 [1.11, 21.33]** |
| Time to the nucleic acid conversion (days) | | | | | | | | | |
| Hu HB 2021 | 100% severe | HJSS | Observational study | 81 | 64.66±8.43 | 43.75 | Cardiovascular disease or coronary heart disease (20.31 %), Diabetes (21.88 %), Hypertension (29.69 %) | 21.03 vs. 23.34 days ^a^ | MD=-2.31 [-5.18, 0.56] |
| Huang DH 2021 | 100% severe | GZ3 | Observational study | 50 | 58.84±5.12 | 60.00 | NR | **17.88 vs.**  **18.76 days** | **MD=-0.88 [-1.39, -0.37]** |
| Qin YL 2020 | 100% severe/critical | RDN | Observational study | 47 | 58.17± 2.97 | 53.19 | Respiratory condition (2.13%), Cardiovascular disease or coronary heart disease (6.38%), Diabetes (29.79%), Hypertension (17.02) | **15.60 vs. 17.70 days** | **MD=-2.10 [-3.15, -1.05]** |
| Wang Y 2021 | 100% severe | HSBD+XYP+XBJ+SM | Observational study | 55 | 55.83±17.89 | 52.70 | Respiratory condition (3.60%), Cardiovascular disease or coronary heart disease (23.60%) | **10.83 vs. 15.50 days** | **MD=-4.67 [-7.41, -1.93]** |

a: Propensity score-matched

**Table S10 Summary of results of rate of conversion to moderate/critical cases**

| **Study** | **Type** | **TCM** | **Design** | **N** | **Mean age (ys)** | **Men (%)** | **Comorbidities** | **Treat vs. control** | **RR (95%CI)** |
| --- | --- | --- | --- | --- | --- | --- | --- | --- | --- |
| Rate of conversion to critical cases | | | | | | | | | |
| Wen L 2020 | 100% severe | XBJ | RCT | 60 | 47.97±5.29 | 53.3 | NR | 0% vs. 5.00% | RR=0.17 [0.01, 4.01] |
| Luo ZJ 2021 | 100% severe | XBJ | RCT | 57 | 58.34±17.30 | NR | Cardiovascular disease or coronary heart disease (5.26%), Diabetes (14.04 %), Hypertension (52.63 %) | **10.34% vs. 35.71%** | **RR=0.29 [0.09, 0.94]** |
| Zhou S 2021 | 32.43% severe, 67.57% critical | SH | RCT | 111 | 64.67±12.14 | 63.96 | NR | **47.4% vs. 88.2%** | **RR=0.54 [0.32, 0.89]** |
| Xiong YB 2022 | 100% severe | HSBD | Observational study | 111 | 61.81 | 67.60 | Respiratory condition (7.20%), Cardiovascular disease or coronary heart disease (44.10%) | 0% vs. 0% | NA |
| Rate of conversion to moderate cases | | | | | | | | | |
| Wen L 2020 | 100% severe | XBJ | RCT | 60 | 47.97±5.29 | 53.3 | NR | 52.50% vs. 40.00% | RR=1.31 [0.71, 2.42] |
| Luo ZJ 2021 | 100% severe | XBJ | RCT | 57 | 58.34±17.30 | NR | Cardiovascular disease or coronary heart disease (5.26%), Diabetes (14.04 %), Hypertension (52.63 %) | 86.21% vs. 64.29% | RR=1.34 [0.98, 1.83] |
| Zhang P 2021 | 75.00% severe, 25.00% critical | QFPD | Observational study | 24 | 62.84 ± 14.69 | 54.17 | NR | 91.67% vs. 58.33% | RR= 1.57 [0.95, 2.61] |

**Table S11 Summary of results of rate/time of symptom resolution**

| **Study** | **Type** | **TCM** | **Design** | **N** | **Mean age (ys)** | **Men (%)** | **Comorbidities** | **Symptom** | **Treat vs. control** | **RR/Mean (95%CI)** |
| --- | --- | --- | --- | --- | --- | --- | --- | --- | --- | --- |
| Rate of symptom resolution | | | | | | | | | | |
| Zhang P 2021 | 75.00% severe, 25.00% critical | QFPD | Observational study | 24 | 62.84 ± 14.69 | 54.17 | NR | Fever  CT improvement | 91.67% vs.75.00%  83.33% vs. 58.33% | RR=3.67 [0.32, 41.59]  RR=1.43 [0.83, 2.45] |
| Huang DH 2021 | 100% severe | GZ3 | Observational study | 50 | 58.84±5.12 | 60.00 | NR | CT improvement | 92.00% vs. 72.00% | RR= 1.28 [0.98, 1.67] |
| Time of symptom resolution | | | | | | | | | | |
| Luo ZJ 2021 | 100% severe | XBJ | RCT | 57 | 58.34±17.30 | NR | Cardiovascular disease or coronary heart disease (5.26%), Diabetes (14.04 %), Hypertension (52.63 %) | Fever  Cough  Shortness of breath  Fatigue | **5.54 vs. 7.34 days**  **7.47 vs. 9.46 days**  **8.36 vs. 10.26 days**  **6.67 vs. 12.16 days** | **MD=-1.80 [-3.03, -0.57]**  **MD=-1.99 [-3.05, -0.93]**  **MD=-1.90 [-3.56, -0.24]**  **MD=-5.49 [-6.92, -4.06]** |
| Hu HB 2021 | 100% severe | HJSS | Observational study | 81 | 64.66±8.43 | 43.75 | Cardiovascular disease or coronary heart disease (20.31 %), Diabetes (21.88 %), Hypertension (29.69 %) | Fever  Cough | 12.53 vs. 15.75 days ^a^  18.06 vs. 20.84 days ^a^ | MD=-3.22 [-6.71, 0.27]  MD=-2.78 [-7.25, 1.69] |
| Wang YY 2021 | 100% severe | CHJD+FZJF | Observational study | 130 | 64.17 | 45.00 | Respiratory condition (37.00%), Cardiovascular disease or coronary heart disease (19.00%), Diabetes (16.00%), Hypertension 41.00%) | Fever  Cough  Dyspnea | **3.67 vs. 7.10 days**  **16.00 vs. 21.00 days**  19.00 vs. 20.00 days | **MD=-3.43 [-4.63, -2.23]**  **MD=-5.00 [-9.19, -0.81]**  MD=-1.00 [-5.12, 3.12] |

a: Propensity score-matched

**Table S12 Summary of results of discharge rate and length of hospital stay**

| **Study** | **Type** | **TCM** | **Design** | **N** | **Mean age (ys)** | **Men (%)** | **Comorbidities** | **Outcome** | **Treat vs. control** | **RR/Mean (95%CI)** |  |
| --- | --- | --- | --- | --- | --- | --- | --- | --- | --- | --- | --- |
| Rate of discharge | | | | | | | | | | |  |
| Feng J 2021 | 100% critical | SH | Observational study | 118 | 66.67±13.66 | 67.10 | Cardiovascular disease or coronary heart disease (17.70%), Diabetes (21.10%), Hypertension 44.00%) | / | **42.40% vs. 17.60%** | **RR= 2.40 [1.31, 4.41]** |  |
| Wang Y 2021 | 100% severe | HSBD+XYP+XBJ+SM | Observational study | 55 | 55.83±17.89 | 52.70 | Respiratory condition (3.60%), Cardiovascular disease or coronary heart disease (23.60%) | / | 86.96% vs. 78.13% | RR= 1.11 [0.87, 1.42] |  |
| Liu XS  2021 | 100% severe/critical | XBJ | Observational study | 144 | 64.60±9.50 | 72.90 | Cardiovascular disease or coronary heart disease (15.30%), Diabetes (24.30%), Hypertension 48.60%) | / | **66.67% vs. 22.22%** | **RR= 3.00 [1.89, 4.76]** |  |
| Wang Y Y  2021 | 100% severe | CHJD+FZJF | Observational study | 130 | 64.17 | 45.00 | Respiratory condition (37.00%), Cardiovascular disease or coronary heart disease (19.00%), Diabetes (16.00%), Hypertension 41.00%) | / | 49.00% vs.56.00% | RR= 1.00 [0.65, 1.54] |  |
| length of hospital/ICU stay | | | | | | | | | | |  |
| Luo ZJ 2021 | 100% severe | XBJ | RCT | 57 | 58.34±17.30 | NR | Cardiovascular disease or coronary heart disease (5.26%), Diabetes (14.04 %), Hypertension (52.63 %) | ICU | **8.4 vs. 10.7 days** | **MD= -2.30 [-3.88, -0.72]** |  |
| Qin YL 2020 | 100% severe/critical | RDN | Observational study | 47 | 58.17± 2.97 | 53.19 | Respiratory condition (2.13%), Cardiovascular disease or coronary heart disease (6.38%), Diabetes (29.79%), Hypertension (17.02) | Hospital  ICU | **17.50 vs. 23.40 days**  **13.90 vs. 18.10 days** | **MD= -5.90 [-6.91, -4.89]**  **MD= -4.20 [-5.00, -3.40]** |  |
| Feng J 2021 | 100% critical | SH | Observational study | 118 | 66.67±13.66 | 67.10 | Cardiovascular disease or coronary heart disease (17.70%), Diabetes (21.10%), Hypertension 44.00%) | ICU | 30.83 vs. 41.00 days | MD= -10.17 [-20.99, 0.65] |  |
| Zhao J  2020 | 100% severe | YDZF | Observational study | 39 | NR | 56.41 | NR | Hospital | 16.00 vs. 17.06 days | MD= -1.06 [-5.28, 3.16] |  |

**Table S13 Summary of results of laboratory markers**

**Biochemical:** Albumin, Alanine aminotransferase (ALT), Aspartate aminotransferase (AST), Total bilirubin (Bili), Blood urea nitrogen, Creatinine, Creatine kinase (CK), Lactate dehydrogenase (LDH), Cardiac troponin I, Myoglobin, Creatine kinase-MB, Glucose, Serum creatinine (Cr), N-terminal proBNP (NT-proBNP), Angiotensin II level.

**Inflammatory biomarkers:** Erythrocyte sedimentation rate (ESR), C-reactive protein (CRP), Serum ferritin, procalcitonin (PCT), IL-1beta, Interleukin-2 receptor (IL-2R), Interleukin-6(IL-6), Interleukin-8(IL-8), Interleukin-10(IL-10), Tumour necrosis factor alpha (TNF-alpha), Interleukin-1 (IL-1), Interleukin-2 (IL-2), Interleukin-7 (IL-7), Interferon-γ induced protein 10 (IP-10), Monocyte chemoattractant protein 1 (MCP-1), Chemokine (C-C Motif) Ligand 3 (CCL-3)

**Coagulation:** Prothrombin time (PT), Activated partial thromboplastin time (APTT), D-dimer, Antithrombin (AT), Thrombin clotting time (TCT), Fibrin degradation products (FDP), Fibrinogen.

**Hematologic:** White blood cell count/Leukocytes count (WBC), Neutrophil count (NC), Lymphocyte count (LYM), CD4, CD8 cell count, Monocyte count, Eosinophil count, Platelet count, Hemoglobin, Thrombocyte count, Granulocyte-colony stimulating factor (G-CSF), NLR(Neutrophils/lymphocytes).

| **Study** | **Type** | **TCM** | **Design** | **N** | **Mean age (ys)** | **Men (%)** | **Comorbidities** | **Outcomes** | | **Intervention / exposure (Difference Mean±SD)** | **Control/ non-exposure (Mean±SD)** | **P value** |
| --- | --- | --- | --- | --- | --- | --- | --- | --- | --- | --- | --- | --- |
|  |  |  |  |  |  |  |  | **Classification** | **Symptom** |  |  |  |
| Wen L | 100% severe | XBJ | RCT | 60 | 47.97±5.29 | 53.3 | NR | Hematologic | WBC(×109/L) | **-1.41±0.53(50ml);**  **-2.63±0.58(100ml)** | **-1.2±0.49** | **P＜0.05** |
|  |  |  |  |  |  |  |  |  | LYM(×109/L) | **-0.2±0.36(50ml);**  **-0.27±0.23(100ml)** | **-0.14±0.29** | **P＜0.05** |
|  |  |  |  |  |  |  |  | Inflammatory biomarkers | CRP (mg/L) | **12.3±4.87(50ml);**  **19.3±5.84(100ml)** | **4.3±5.76** | **P＜0.05** |
|  |  |  |  |  |  |  |  |  | ESR(mm/1h) | **5.2±6.08(50ml);**  **18.6±6.92(100ml)** | **1.6±6.86** | **P＜0.05** |
| Liu YJ | 100% severe | HSBD | RCT | 50 | 48.25±1.49 | 54 | NR | Hematologic | *WBC(×109/L) | **7.60±0.60** | **5.50±0.50** | **P＜0.05** |
|  |  |  |  |  |  |  |  |  | *LYM(×109/L) | **2.30±0.70** | **1.00±0.10** | **P＜0.05** |
|  |  |  |  |  |  |  |  | Inflammatory biomarkers | *CRP (mg/L) | **27.10±5.10** | **37.60±6.00** | **P＜0.05** |
|  |  |  |  |  |  |  |  |  | *ESR(mm/1h) | **40.00±6.40** | **55.90±5.70** | **P＜0.05** |
| Hu F | 100% severe | CS | RCT | 40 | 61.17±15.38 | 60 | Respiratory condition (2.50%), Cardiovascular disease or coronary heart disease (7.50%), Diabetes (22.50%), Hypertension (30.00%) | Biochemical | AST (U/L) | 8.22±21.29 | 14.78 ±25.78 | P=0.38 |
|  |  |  |  |  |  |  |  |  | ALT (U/L) | 2.16±30.22 | -10.68±34.59 | P=0.21 |
|  |  |  |  |  |  |  |  |  | TB (U/L) | -0.42±6.69 | -1.19±5.69 | P=0.70 |
|  |  |  |  |  |  |  |  |  | Cr (mmol/L) | 4.34±18.07 | 8.57±31.81 | P=0.60 |
|  |  |  |  |  |  |  |  |  | CK-MB (U/L) | 14.25±60.42 | 1.38±10.09 | P=0.36 |
|  |  |  |  |  |  |  |  | Hematologic | PLT (U/L) | -7.00±60.13 | -27.10±82.88 | P=0.38 |
|  |  |  |  |  |  |  |  |  | ROX | **-5.25±3.72** | **-1.30±4.91** | **P＜0.05** |
|  |  |  |  |  |  |  |  |  | WBC(×10^9^/L) | 1.76±4.01 | 1.53±2.39 | P=0.83 |
|  |  |  |  |  |  |  |  |  | PBML(peripheral blood mononuclear lymphocyte) (10^9^/L) | 0.13±0.47 | 0.44±0.58 | P=0.06 |
| Luo ZJ | 100% severe | XBJ | RCT | 57 | 58.34±17.30 | NR | Cardiovascular disease or coronary heart disease (5.26%), Diabetes (14.04 %), Hypertension (52.63 %) | Inflammatory biomarkers | IL-6 (ng/ml) | **0.03±0.01** | **0.02±0.01** | **P＜0.05** |
|  |  |  |  |  |  |  |  |  | IL-8 (ng/ml) | **0.12±0.15** | **0.04 ±0.15** | **P＜0.05** |
|  |  |  |  |  |  |  |  |  | TNF-𝛼 (pg/ml) | **18.01±10.74** | **3.59±10.82** | **P＜0.05** |
|  |  |  |  |  |  |  |  |  | CRP (mg/L) | 31.52±41.95 | 22.45±20.19 | P=0.30 |
|  |  |  |  |  |  |  |  | Hematologic | LYM (×10^9^/L) | **-0.39±0.50** | **0.04 ±0.39** | **P＜0.05** |
| ^†^Hu HB | 100% severe | HJSS | Observational study | 81 | 64.66±8.43 | 43.75 | Cardiovascular disease or coronary heart disease (20.31 %), Diabetes (21.88 %), Hypertension (29.69 %) | Hematologic | *SpO_2_ (%) | 10.91±2.4 | 10.34±2.15 | P=0.33 |
|  |  |  |  |  |  |  |  |  | *LYM (×10^9^/L) | 0.5±0.29 | 0.43±0.24 | P=0.27 |
|  |  |  |  |  |  |  |  |  | *WBC (×10^9^/L) | **-0.57±2.55** | **1.38±2.99** | **P=0.01** |
|  |  |  |  |  |  |  |  |  | *NC (×10^9^/L) | -0.92 ±1.85 | -0.22 ±2.51 | P=0.22 |
|  |  |  |  |  |  |  |  | Inflammatory biomarkers | *CRP (mg/L) | 17.44±5.92 | 17.62±6.54 | P=0.91 |
| Huang DH | 100% severe | GZ 3 | Observational study | 50 | 58.84±5.12 | 60 | NR | Hematologic | NC(×10^9^/L) | **4.65±1.34** | **2.45±1.34** | **P＜0.05** |
|  |  |  |  |  |  |  |  |  | LYM(×10^9^/L) | **-0.89±0.23** | **-0.51±0.35** | **P＜0.05** |
|  |  |  |  |  |  |  |  |  | NLR(Neutrophils/lymphocytes) | **7.67±2.58** | **5.13±2.05** | **P＜0.05** |
| Zhang P | 75% Severe and 25% Critical | QFPD | Observational study | 24 | 62.84 ± 14.69 | 54.17 | NR | Inflammatory biomarkers | IL-2R | 588.16±438.54 | 287.09±440.82 | P=0.09 |
|  |  |  |  |  |  |  |  |  | IL-6 | 42.16±77.08 | 52.18 ±80.92 | P=0.76 |
|  |  |  |  |  |  |  |  |  | IL-8 | 20.62 ±19.40 | 10.71 ±15.80 | P=0.17 |
|  |  |  |  |  |  |  |  |  | IL-10 | 3.39 ±9.14 | 0.62 ±1.46 | P=0.30 |
|  |  |  |  |  |  |  |  |  | TNF-α | 4.59 ±4.43 | 1.76±3.64 | P=0.09 |
|  |  |  |  |  |  |  |  |  | PCT | 0.03±0.04 | 0.06±0.25 | P=0.68 |
|  |  |  |  |  |  |  |  |  | CRP | 34.95±48.30 | 44.53±53.02 | P=0.64 |
|  |  |  |  |  |  |  |  | Hematologic | WBC | 0.32 ±1.67 | -0.03 ±3.71 | P=0.77 |
|  |  |  |  |  |  |  |  |  | NC | 0.71 ±1.43 | 0.52±3.71 | P=0.87 |
|  |  |  |  |  |  |  |  |  | LYM | **-0.09±0.45** | **-0.46±0.40** | **P＜0.05** |
| Qin YL | 100% severe/critical | RDN | Observational study | 47 | 58.17±2.97 | 53.19 | Respiratory condition (2.13 %), Cardiovascular disease or coronary heart disease (6.38 %), Diabetes (29.79 %), Hypertension (17.02 %) | Hematologic | WBC (×10^9^/L) | **1.23±0.69** | **-2.59±0.65** | **P＜0.05** |
|  |  |  |  |  |  |  |  |  | Neutrophil (%) | **9.20 ±2.82** | **6.30±3.47** | **P＜0.05** |
|  |  |  |  |  |  |  |  |  | LYM (×109/L) | **-0.21±0.12** | **-0.28±0.09** | **P＜0.05** |
|  |  |  |  |  |  |  |  |  | Lymphocyte (%) | **-4.60±2.34** | **1.50±1.73** | **P＜0.05** |
|  |  |  |  |  |  |  |  |  | CD4^+^ T cells (mm3) | **-186.10±64.00** | **-91.90±43.95** | **P＜0.05** |
|  |  |  |  |  |  |  |  |  | CD8^+^ T cells (mm3) | **-181.30±105.74** | **-80.70±42.44** | **P＜0.05** |
|  |  |  |  |  |  |  |  |  | CD4^+^/CD8^+^ | -0.54 ±0.54 | -0.33±0.23 | P=0.10 |
|  |  |  |  |  |  |  |  |  | Total T cells (mm3) | **-419.70 ±109.83** | **-80.80±78.45** | **P＜0.05** |
|  |  |  |  |  |  |  |  | Inflammatory biomarkers | PCT (ng/mL) | **0.07±0.10** | **-0.04±0.13** | **P＜0.05** |
|  |  |  |  |  |  |  |  |  | CRP (mg/L) | **92.40 ±12.04** | **-1.10 ±17.51** | **P＜0.05** |
|  |  |  |  |  |  |  |  |  | IL-4(pg/mL) | **-1.14 ±0.34** | **-0.07 ±0.29** | **P＜0.05** |
|  |  |  |  |  |  |  |  |  | IL-6(pg/mL) | **25.36±7.54** | **-33.49±22.36** | **P＜0.05** |
|  |  |  |  |  |  |  |  |  | IL-10(pg/mL) | **-1.01 ±1.11** | **-11.23±7.28** | **P＜0.05** |
|  |  |  |  |  |  |  |  |  | IL-17(pg/mL) | **-0.33±0.30** | **-8.84±14.76** | **P＜0.05** |
|  |  |  |  |  |  |  |  |  | TNF－α(ng/mL) | **-2.56±0.88** | **0.34±0.66** | **P＜0.05** |
|  |  |  |  |  |  |  |  |  | IFN－γ(ng/mL) | **-1.62±6.59** | **2.07±3.51** | **P＜0.05** |
|  |  |  |  |  |  |  |  | Biochemical | LDH (U/L) | 140.17 ±169.37 | 109.67±119.60 | P=0.49 |
| Wang Y | 100%Severe | HSBD+XYP+XBJ+SM+Supportive therapy | Observational study | 55 | 55.83±17.89 | 52.7 | Respiratory condition (3.6%)，Cardiovascular disease or coronary heart disease (23.6%) | Hematologic | WBC(×10^9^/L) | -0.81 ±1.74 | 0.47 ±1.62 | P >0.05 |
|  |  |  |  |  |  |  |  |  | LYM (×10^9^/L) | -0.32 ±0.55 | -0.22 ±0.53 | P >0.05 |
|  |  |  |  |  |  |  |  |  | Lymphocyte (%) | -3.30 ±9.09 | -5.44 ±8.18 | P >0.05 |
|  |  |  |  |  |  |  |  |  | NLR(Neutrophils/lymphocytes) | 0.71 ±1.56 | 1.23 ±1.94 | P >0.05 |
|  |  |  |  |  |  |  |  |  | Ferroprotein (ng/mL) | **156.20±342.05** | **29.77±295.98** | P **<0.05** |
|  |  |  |  |  |  |  |  | Inflammatory biomarkers | ESR (mm/h) | 12.58 ±22.45 | 9.76 ±22.65 | P >0.05 |
|  |  |  |  |  |  |  |  |  | High sensitivity CRP (mg/L) | **21.38 ±37.66** | **9.37 ±22.80** | P **<0.05** |
|  |  |  |  |  |  |  |  |  | IL-6 (pg/mL) | 0.91±4.06 | -0.03±3.73 | P >0.05 |
|  |  |  |  |  |  |  |  | Coagulation | D dimer (ug/mL) | -0.03±0.44 | -0.09±0.50 | P >0.05 |
|  |  |  |  |  |  |  |  | Biochemical | AST(U/L) | 9.67±16.46 | 8.08±13.03 | P >0.05 |
|  |  |  |  |  |  |  |  |  | ALT (U/L) | -1.67±22.61 | 3.33±25.23 | P >0.05 |
|  |  |  |  |  |  |  |  |  | CK (U/L) | 37.33±47.74 | 12.67±28.60 | P >0.05 |
|  |  |  |  |  |  |  |  |  | Hypersensitive troponin (pg/mL) | 1.68±4.18 | 0.08±2.04 | P >0.05 |
|  |  |  |  |  |  |  |  |  | Myohemoglobin (ng/mL) | 10.07±24.30 | 1.80±14.03 | P >0.05 |
|  |  |  |  |  |  |  |  |  | Lactate dehydrogenase (U/L) | 55.50±87.35 | 61.08±77.84 | P >0.05 |
| Chen L | 100% severe/critical | XBJ | Observational study | 65 | 61.89±13.51 | 50.77 | NR | Hematologic | WBC(×109/L) | -0.34±3.02 | -0.62±3.33 | P=0.75 |
|  |  |  |  |  |  |  |  |  | Neutrophil (%) | 0.00±0.11 | 0.04±0.15 | P=0.28 |
|  |  |  |  |  |  |  |  |  | Lymphocyte (%) | 0.01±0.09 | 0.02±0.10 | P=0.70 |
|  |  |  |  |  |  |  |  |  | PLT(×109/L) | **-32.82±132.84** | **76.10±109.28** | **P＜0.05** |
|  |  |  |  |  |  |  |  |  | T lymphocyte counts(×10^9^/L) | **-226.44±338.32** | **-32.69±239.36** | **P＜0.05** |
|  |  |  |  |  |  |  |  |  | CD4^+^ T cells (%) | -0.09±0.13 | -0.05±0.15 | P=0.30 |
|  |  |  |  |  |  |  |  |  | CD4 ^+^ T cell count(×10^9^/L) | **-149.11±195.56** | **-11.04±158.26** | **P＜0.05** |
|  |  |  |  |  |  |  |  |  | CD8^+^ T cells (%) | 0.00±0.14 | -0.01±0.12 | P=0.77 |
|  |  |  |  |  |  |  |  |  | CD8+ T cell count (×109/L) | -41.33±147.73 | -10.67±80.56 | P=0.28 |
|  |  |  |  |  |  |  |  |  | CD4+/CD8+ | **-3.38±3.23** | **0.13±0.75** | **P＜0.05** |
|  |  |  |  |  |  |  |  |  | PaO2(mmHg) | -33.06±24.36 | -21.85±34.61 | P=0.19 |
|  |  |  |  |  |  |  |  |  | FiO2 | **0.04±0.17** | **-0.05±0.08** | **P＜0.05** |
|  |  |  |  |  |  |  |  | Biochemical | CK(U/L) | 51.16±110.08 | 67.10±92.90 | P=0.55 |
|  |  |  |  |  |  |  |  |  | CK-MB(U/L) | 0.60±5.26 | 1.67±4.38 | P=0.39 |
|  |  |  |  |  |  |  |  |  | TBil(μmol/L) | -1.76±7.87 | -0.56±6.99 | P=0.54 |
|  |  |  |  |  |  |  |  |  | ALT(U/L) | **-15.80±38.95** | **1.17±13.98** | **P＜0.05** |
|  |  |  |  |  |  |  |  |  | AST(U/L) | **4.62±23.85** | **-7.93±13.17** | **P＜0.05** |
|  |  |  |  |  |  |  |  |  | ALB(g/L) | 2.94±4.699 | 1.70±6.53 | P=0.44 |
|  |  |  |  |  |  |  |  |  | LDH(μmol·s-1·L-1) | 0.86±1.75 | 0.60±0.95 | P=0.44 |
|  |  |  |  |  |  |  |  |  | BUN (mmol/L) | -0.16±2.64 | -0.13±1.74 | P=0.96 |
|  |  |  |  |  |  |  |  |  | Serum creatinine(μmol/L) | -1.76±41.96 | 1.70±19.19 | P=0.65 |
|  |  |  |  |  |  |  |  | Inflammatory biomarkers | PCT(μg/L) | 0.01±0.11 | -0.01±0.03 | P=0.26 |
|  |  |  |  |  |  |  |  |  | IL-4(ng/L) | -0.71±1.15 | -0.30±1.49 | P=0.27 |
|  |  |  |  |  |  |  |  |  | IL-6(ng/L) | 13.27±54.64 | 0.80±13.73 | P=0.15 |
|  |  |  |  |  |  |  |  |  | IL-10(ng/L) | -0.06±3.56 | 1.07±2.11 | P=0.11 |
|  |  |  |  |  |  |  |  |  | IL-17(ng/L) | -0.28±0.42 | -4.90±18.94 | P=0.28 |
|  |  |  |  |  |  |  |  |  | IFN-γ(ng/L) | -0.80±7.16 | 0.84±3.92 | P=0.24 |
|  |  |  |  |  |  |  |  |  | TNF-α(ng/L) | -1.72±4.57 | -1.44±4.92 | P=0.83 |
| ^†^Sun QG | 61.20% severe, 38.8% critical | TCMDs | Observational study | 282 | 68.67±13.45 | 55.90 | Cardiovascular disease or coronary heart disease (16.5%), Diabetes (19.10%), Hypertension 48.40%) | Biochemical | ALB | -0.87±5.30 | 0.30±5.64 | P=0.14 |
|  |  |  |  |  |  |  |  |  | ALT | -3.00±22.92 | -3.67±20.22 | P=0.83 |
|  |  |  |  |  |  |  |  |  | AST | 7.33±17.22 | 5.00±19.21 | P=0.38 |
|  |  |  |  |  |  |  |  |  | BUN | -1.23±4.12 | -1.07±8.66 | P=0.87 |
|  |  |  |  |  |  |  |  |  | Cr | **-0.13±32.46** | **-58.90±200.04** | **P＜0.05** |
|  |  |  |  |  |  |  |  |  | Uric acid | 23.50±104.21 | 30.67±162.10 | P=0.72 |
|  |  |  |  |  |  |  |  |  | CK | **53.67±83.57** | **19.00±99.93** | **P＜0.05** |
|  |  |  |  |  |  |  |  |  | Potassium | -0.30±0.60 | -0.22±0.66 | P=0.38 |
|  |  |  |  |  |  |  |  |  | Chlorine | -0.33±4.19 | -1.33±4.94 | P=0.13 |
|  |  |  |  |  |  |  |  |  | Sodium | **-0.70±5.21** | **-2.50±5.27** | **P＜0.05** |
|  |  |  |  |  |  |  |  |  | Blood sugar | 0.51±2.15 | -0.14±2.72 | P=0.07 |
|  |  |  |  |  |  |  |  | Inflammatory biomarkers | CRP | 44.23±76.90 | 31.16±72.53 | P=0.23 |
|  |  |  |  |  |  |  |  | Hematologic | PLT | **-8.57±96.21** | **21.17±84.18** | **P＜0.05** |
|  |  |  |  |  |  |  |  |  | Red blood cell | 0.23±0.69 | 0.32±0.83 | P=0.42 |
|  |  |  |  |  |  |  |  |  | WBC | 0.08±2.93 | -0.17±2.54 | P=0.53 |
|  |  |  |  |  |  |  |  |  | Percentage of monocytes | 0.20±2.83 | 0.07±3.88 | P=0.79 |
|  |  |  |  |  |  |  |  |  | Percentage of lymphocytes | -3.53±12.50 | -3.03±14.14 | P=0.80 |
|  |  |  |  |  |  |  |  |  | Percentage of basophils | **-1.15±2.02** | **-0.50±1.17** | **P＜0.05** |
|  |  |  |  |  |  |  |  |  | Percentage of eosinophils | 0.15±0.29 | 0.09±0.14 | P=0.07 |
|  |  |  |  |  |  |  |  |  | Hemoglobin | 9.00±19.92 | 8.33±22.22 | P=0.83 |
|  |  |  |  |  |  |  |  |  | Percentage of neutrophils | 4.50±16.38 | 3.28±19.13 | P=0.64 |
| Zhao J | 100% severe | YDZF | Observational study | 39 | NR | 56.41 | NR | Inflammatory biomarkers | TNF-α* | **6.61±2.37** | **8.75±3.28** | **P=0.035** |
|  |  |  |  |  |  |  |  |  | IL-6* | **2.9±2.81** | **7.67±8.71** | **P=0.013** |
|  |  |  |  |  |  |  |  |  | IL-8* | 2.5±0 | 3.14±1.51 | P=0.818 |
|  |  |  |  |  |  |  |  |  | IL-2R* | 316.67±179.12 | 462.17±259.66 | P=0.071 |
|  |  |  |  |  |  |  |  |  | CRP* | 10.23±25.68 | 8.48±15.82 | P=0.795 |
|  |  |  |  |  |  |  |  | Hematologic | WBC | 1.37±0.78 | 1.04±1.06 | P=0.908 |
|  |  |  |  |  |  |  |  |  | LYM | 0.22±0.34 | 0.22±0.39 | P=0.884 |
|  |  |  |  |  |  |  |  |  | CD4* | 546.95±280.54 | 604.67±227.80 | P=0.607 |
|  |  |  |  |  |  |  |  |  | CD8* | 342.83±175.34 | 267.33±148.04 | P=0.323 |

**Table S14 Summary of results of PaO2/FiO2**

| **Study** | **Type** | **TCM** | **Design** | **N** | Mean age (ys) | **Men (%)** | **Comorbidities** | **Outcome** | **Treat vs. control** | **Mean (95%CI)** |
| --- | --- | --- | --- | --- | --- | --- | --- | --- | --- | --- |
| Time for relieving blood oxygen saturation | | | | | | | | | | |
| Xiong YB 2022 | 100% severe | HSBD | Observational study | 111 | 61.81 | 67.60 | Respiratory condition (7.20%), Cardiovascular disease or coronary heart disease (44.10%) |  | **6.67 vs. 11.08 days** | **MD=-4.41 [-6.36, -2.46]** |
| PaO2/FiO2 | | | | | | | | | | |
| Hu F 2020 | 100% severe | CS | RCT | 40 | 61.17±15.38 | 60.00 | Respiratory condition (2.50%), Cardiovascular disease or coronary heart disease (7.50%), Diabetes (22.50%), Hypertension (30.00%) | PaO_2_/FiO_2_ | **73.60 vs. 14.92^a^ mmHg** | **MD=58.68 [24.86, 92.50]** |
| Hu HB 2021 | 100% severe | HJSS | Observational study | 81 | 64.66±8.43 | 43.75 | Cardiovascular disease or coronary heart disease (20.31 %), Diabetes (21.88 %), Hypertension (29.69 %) | PaO_2_/FiO_2_ | 100.25 vs. 100.81^b^ mmHg | MD=0.22 [-9.56, 10.00] |
| Chen L  2021 | 100% severe/critical | XBJ | Observational study | 65 | 61.89 | 50.77 | NR | PaO_2_/FiO_2_ | **85.78 vs. 35.11 ^a^ mmHg** | **MD=120.89 [71.41, 170.37]** |
| Liu XS  2021 | 100% severe/critical | XBJ | Observational study | 144 | 64.60±9.50 | 72.90 | Cardiovascular disease or coronary heart disease (15.30%), Diabetes (24.30%), Hypertension 48.60%) | PaO_2_/FiO_2_ | \| **110.80 vs.** \| \| --- \| \| **25.80 ^a^ mmHg** \| | **MD=136.60 [104.73, 168.47]** |

a: After treatment minus before treatment

b: Propensity score-matched

**Table S15 Incidence of adverse reactions**

| **TCM** | **Study Design** | **No. of Participations (I/C)** | **Adverse Reactions** | **Intervention Group** | | **Control Group** | | **P Value** |
| --- | --- | --- | --- | --- | --- | --- | --- | --- |
|  |  |  |  | Event | % | Event | % |  |
| HSBD | RCT | 50 (25/25) | Gastrointestinal bleeding | 1 | 4.00% | 2 | 8.00% | **<0.05** |
|  |  |  | Prolonged coagulation time | 0 | 0.00% | 1 | 4.00% | **<0.05** |
|  |  |  | Total | 1 | 4.00% | 3 | 12.00% | **<0.05** |
| CS | RCT | 40 (21/19) | Total | 0 | 0.00% | 0 | 0.00% | NA |
| XBJ | RCT | 57 (29/28) | Abnormal liver function | 5 | 17.24% | 3 | 10.71% | 0.348 |
|  |  |  | Renal dysfunction | 3 | 10.34% | 4 | 14.29% | 0.765 |
|  |  |  | Rash | 2 | 6.90% | 1 | 3.57% | 0.554 |
|  |  |  | Anaphylactic shock | 0 | 0.00% | 0 | 0.00% | NA |
|  |  |  | Acute respiratory distress syndrome | 4 | 13.79% | 12 | 42.86% | **0.042** |
|  |  |  | Septic shock | 2 | 6.90% | 8 | 28.57% | **0.026** |
|  | Observational study | 144 (72/72) | Total | 30 | 41.67% | 31 | 43.06% | 0.866 |
| SH | RCT | 111 (54/57) | Hypoalbuminemia | 21 | 38.89% | 38 | 66.67% | **<0.001** |
|  |  |  | Hypokalemia | 4 | 7.41% | 9 | 15.79% | 0.114 |
|  |  |  | Increased blood glucose | 30 | 55.56% | 43 | 75.44% | **0.003** |
|  |  |  | Anemia | 29 | 53.70% | 29 | 50.88% | 0.766 |
|  |  |  | Rash | 1 | 1.85% | 0 | 0.00% | 1.000 |
|  |  |  | Thrombocytopenia | 21 | 38.89% | 36 | 63.16% | **0.002** |
|  |  |  | Increased total bilirubin | 7 | 12.96% | 19 | 33.33% | **0.004** |
|  |  |  | Increased blood lipids | 19 | 35.19% | 24 | 42.11% | 0.23 |
|  |  |  | Increased white cell count | 23 | 42.59% | 43 | 75.44% | **<0.001** |
|  |  |  | Increased blood urea nitrogen | 18 | 33.33% | 31 | 54.39% | **0.006** |
|  |  |  | Increased neutrophil | 30 | 55.56% | 49 | 85.96% | **<0.001** |
|  |  |  | Increased aspartate aminotransferase | 23 | 42.59% | 35 | 61.40% | **0.01** |
|  |  |  | Constipation | 2 | 3.70% | 0 | 0.00% | 0.496 |
|  |  |  | Nausea | 1 | 1.85% | 4 | 7.02% | 0.198 |
|  |  |  | Vomiting | 1 | 1.85% | 4 | 7.02% | 0.198 |
|  |  |  | Diarrhea | 2 | 3.70% | 9 | 15.79% | **0.02** |
|  |  |  | Abnormal serum sodium | 7 | 12.96% | 29 | 50.88% | <0.001 |
|  |  |  | Increased serum potassium | 10 | 18.52% | 27 | 47.37% | **<0.001** |
|  |  |  | Respiratory failure or acute respiratory distress syndrome | 23 | 42.59% | 43 | 75.44% | **<0.001** |
|  |  |  | Cardiopulmonary failure | 8 | 14.81% | 31 | 54.39% | **<0.001** |
|  |  |  | Pulmonary embolism | 0 | 0.00% | 2 | 3.51% | 0.234 |
|  |  |  | Cardiac arrest | 16 | 29.63% | 30 | 52.63% | **0.003** |
|  |  |  | Acute coronary syndrome | 1 | 1.85% | 1 | 1.75% | 1.000 |
|  |  |  | Tachycardia | 6 | 11.11% | 6 | 10.53% | 0.920 |
|  |  |  | Septic shock | 6 | 11.11% | 5 | 8.77% | 0.823 |
|  |  |  | Sepsis | 7 | 12.96% | 4 | 7.02% | 0.39 |
|  |  |  | Bronchitis | 4 | 7.41% | 1 | 1.75% | 0.364 |
|  |  |  | Thrombocytopenia | 15 | 27.78% | 34 | 59.65% | **<0.001** |
|  |  |  | Increased D-dimer | 43 | 79.63% | 51 | 89.47% | **0.005** |
|  |  |  | Hemorrhage of lower digestive tract | 4 | 7.41% | 0 | 0.00% | 0.119 |
|  |  |  | Acute kidney injury | 7 | 12.96% | 12 | 21.05% | 0.165 |
|  |  |  | Multiple organ dysfunction syndrome | 9 | 16.67% | 28 | 49.12% | **<0.001** |
| HSBD+XYP+XBJ+SM | Observational study | 55 (23/32) | Mild elevation of alanine aminotransferase | 2 | 8.70% | 4 | 12.50% | 0.66 |
|  |  |  | Increased aspartate aminotransferase | 1 | 4.35% | 1 | 3.13% | 0.81 |
| QFPD | Observational study | 8939 (2568/6371) | Acute hepatic injury | 0.96 [0.81, 1.14] ^#^ | | | | 0.658 |
|  |  |  | Kidney injury | 0.85 [0.62, 1.17] ^#^ | | | | 0.318 |
| CHJD+FZJF | Observational study | 86 (43/43) | Increased D-dimer | 14 | 32.56% | 5 | 11.63% | **0.036** |
|  |  |  | Dyspnea | 9 | 20.93% | 5 | 11.63% | 0.382 |
|  |  |  | Fever | 4 | 9.30% | 3 | 6.98% | 1.000 |
|  |  |  | Increased alanine aminotransferase | 5 | 11.63% | 1 | 2.33% | 0.202 |
|  |  |  | Diarrhea | 4 | 9.30% | 2 | 4.65% | 0.676 |
|  |  |  | Increased aspartate aminotransferase | 4 | 9.30% | 1 | 2.33% | 0.360 |
|  |  |  | Decreased peripheral oxygen saturation | 0 | 0.00% | 4 | 9.30% | 0.116 |
|  |  |  | Constipation | 3 | 6.98% | 1 | 2.33% | 0.616 |
|  |  |  | Chest tightness | 3 | 6.98% | 0 | 0.00% | 0.241 |
|  |  |  | Cough | 3 | 6.98% | 0 | 0.00% | 0.241 |
|  |  |  | Rash | 3 | 6.98% | 0 | 0.00% | 0.241 |
|  |  |  | Increased neutrophil | 0 | 0.00% | 3 | 6.98% | 0.241 |
|  |  |  | Increased white cell count | 1 | 2.33% | 1 | 2.33% | 1.000 |
|  |  |  | Thrombocytopenia | 1 | 2.33% | 1 | 2.33% | 1.000 |
|  |  |  | Melena | 1 | 2.33% | 1 | 2.33% | 1.000 |
|  |  |  | Fatigue | 1 | 2.33% | 1 | 2.33% | 1.000 |
|  |  |  | Sleep disorders | 1 | 2.33% | 1 | 2.33% | 1.000 |
|  |  |  | Urination disorder | 1 | 2.33% | 1 | 2.33% | 1.000 |
|  |  |  | Palpitation | 2 | 4.65% | 0 | 0.00% | 0.494 |
|  |  |  | Chest pain | 2 | 4.65% | 0 | 0.00% | 0.494 |
|  |  |  | Pruritus | 2 | 4.65% | 0 | 0.00% | 0.494 |
|  |  |  | Dysphoria | 0 | 0.00% | 2 | 4.65% | 0.494 |
|  |  |  | Hemoptysis | 1 | 2.33% | 0 | 0.00% | 1.000 |
|  |  |  | Nausea and vomiting | 1 | 2.33% | 0 | 0.00% | 1.000 |
|  |  |  | Abdominal discomfort | 1 | 2.33% | 0 | 0.00% | 1.000 |
|  |  |  | Anorexia | 1 | 2.33% | 0 | 0.00% | 1.000 |
|  |  |  | Headache and ophthalmodynia | 1 | 2.33% | 0 | 0.00% | 1.000 |
|  |  |  | Oral ulcer | 1 | 2.33% | 0 | 0.00% | 1.000 |
|  |  |  | Lymphopenia | 0 | 0.00% | 1 | 2.33% | 1.000 |
|  |  |  | Increased C-reactive protein | 0 | 0.00% | 1 | 2.33% | 1.000 |
|  |  |  | Increased NTproBNP | 0 | 0.00% | 1 | 2.33% | 1.000 |
|  |  |  | Atrial fibrillation | 0 | 0.00% | 1 | 2.33% | 1.000 |
|  |  |  | Hypoalbuminemia | 0 | 0.00% | 1 | 2.33% | 1.000 |
|  |  |  | Hyponatremia | 0 | 0.00% | 1 | 2.33% | 1.000 |
|  |  |  | Increased glutamyl transpeptidase | 0 | 0.00% | 1 | 2.33% | 1.000 |
|  |  |  | Increased creatinine | 0 | 0.00% | 1 | 2.33% | 1.000 |
|  |  |  | Increased creatine kinase | 0 | 0.00% | 1 | 2.33% | 1.000 |
|  |  |  | Increased lactate dehydrogenase | 0 | 0.00% | 1 | 2.33% | 1.000 |
|  |  |  | Increased urea nitrogen | 0 | 0.00% | 1 | 2.33% | 1.000 |
|  |  |  | Costalgia | 0 | 0.00% | 1 | 2.33% | 1.000 |
|  |  |  | Respiratory failure | 2 | 4.65% | 0 | 0.00% | 0.494 |
|  |  |  | Shock | 1 | 2.33% | 0 | 0.00% | 1.000 |
|  |  |  | Severe metabolic acidosis | 1 | 2.33% | 0 | 0.00% | 1.000 |
|  |  |  | Unconsciousness | 0 | 0.00% | 2 | 4.65% | 0.494 |
